# Sulfite oxidase deficiency causes persulfidation loss and hydrogen sulfide release

**DOI:** 10.1172/JCI181299

**Published:** 2025-11-03

**Authors:** Chun-Yu Fu, Joshua B. Kohl, Filip Liebsch, Davide D’Andrea, Tamás Ditrói, Seiryo Ogata, Franziska Neuser, Max Mai, Anna T. Mellis, Emilia Kouroussis, Masanobu Morita, Titus Gehling, José Angel Santamaria-Araujo, Sin Yuin Yeo, Heike Endepols, Michaela Křížková, Viktor Kozich, Marcus Krueger, Julia B. Hennermann, Uladzimir Barayeu, Takaaki Akaike, Peter Nagy, Milos Filipovic, Guenter Schwarz

**Affiliations:** 1 Institute of Biochemistry, Department of Chemistry and Biochemistry, University of Cologne, Cologne, Germany.; 2 Leibniz Institute for Analytical Sciences, ISAS e.V., Dortmund, Germany.; 3 Department of Molecular Immunology and Toxicology and the National Tumor Biology Laboratory, National Institute of Oncology, Budapest, Hungary.; 4 Department of Environmental Medicine and Molecular Toxicology, Tohoku University Graduate School of Medicine, Sendai, Japan.; 5 Institute of Diagnostic and Interventional Radiology,; 6 Institute of Radiochemistry and Experimental Molecular Imaging and; 7 Department of Nuclear Medicine, University of Cologne, Faculty of Medicine, and University Hospital Cologne, Cologne, Germany.; 8 Forschungszentrum Jülich GmbH, Institute of Neuroscience and Medicine, Nuclear Chemistry (INM-5), Jülich, Germany.; 9 Department of Pediatrics and Inherited Metabolic Disorders, Charles University, First Faculty of Medicine, and General University Hospital in Prague, Prague, Czech Republic.; 10 Institute of Genetics, Department of Biology and CECAD, University of Cologne, Cologne, Germany.; 11 Center for Pediatric and Adolescent Medicine, University Medical Center Mainz, Mainz, Germany.; 12 Max-Planck-Institute for Polymer Research, Mainz, Germany.; 13 Department of Anatomy and Histology, Hungarian Research Network–UVMB Laboratory of Redox Biology Research Group, University of Veterinary Medicine, Budapest, Hungary.; 14 Chemistry Institute, University of Debrecen, Debrecen, Hungary.; 15 School of Molecular Biosciences, University of Glasgow, Glasgow, United Kingdom.; 16 Center for Molecular Medicine Cologne, University of Cologne, Cologne, Germany.

**Keywords:** Clinical Research, Metabolism, Amino acid metabolism, Mitochondria, Neurodegeneration

## Abstract

Sulfite oxidase (SOX) deficiency is a rare inborn error of cysteine metabolism resulting in severe neurological damage. In patients, sulfite accumulates to toxic levels, causing a rise in the downstream products *S*-sulfocysteine, which mediates excitotoxicity, and thiosulfate, a catabolic intermediate/product of hydrogen sulfide (H_2_S) metabolism. Here, we report a full-body knockout mouse model for SOX deficiency (SOXD) with a severely impaired phenotype. Among the urinary biomarkers, thiosulfate showed a 45-fold accumulation in SOXD mice, representing the major excreted S-metabolite. Consistently, we found increased plasma H_2_S, which was derived from sulfite-induced release from persulfides, as demonstrated in vitro and in vivo. Mass spectrometry analysis of total protein persulfidome identified a major loss of S-persulfidation in 20% of the proteome, affecting enzymes in amino acids, fatty acid metabolism, and cytosolic iron-sulfur cluster biogenesis. Urinary amino acid profiles indicated metabolic rewiring and mitochondrial dysfunction, thus identifying an altered H_2_S metabolism and persulfidation in SOXD. Finally, oxidized glutathione and glutathione trisulfide were able to scavenge sulfite in vitro and in vivo, extending the lifespan of SOXD mice and providing a mechanistic concept of sulfite scavenging for the treatment of this severe metabolic disorder of cysteine catabolism.

## Introduction

**Figure 1:**
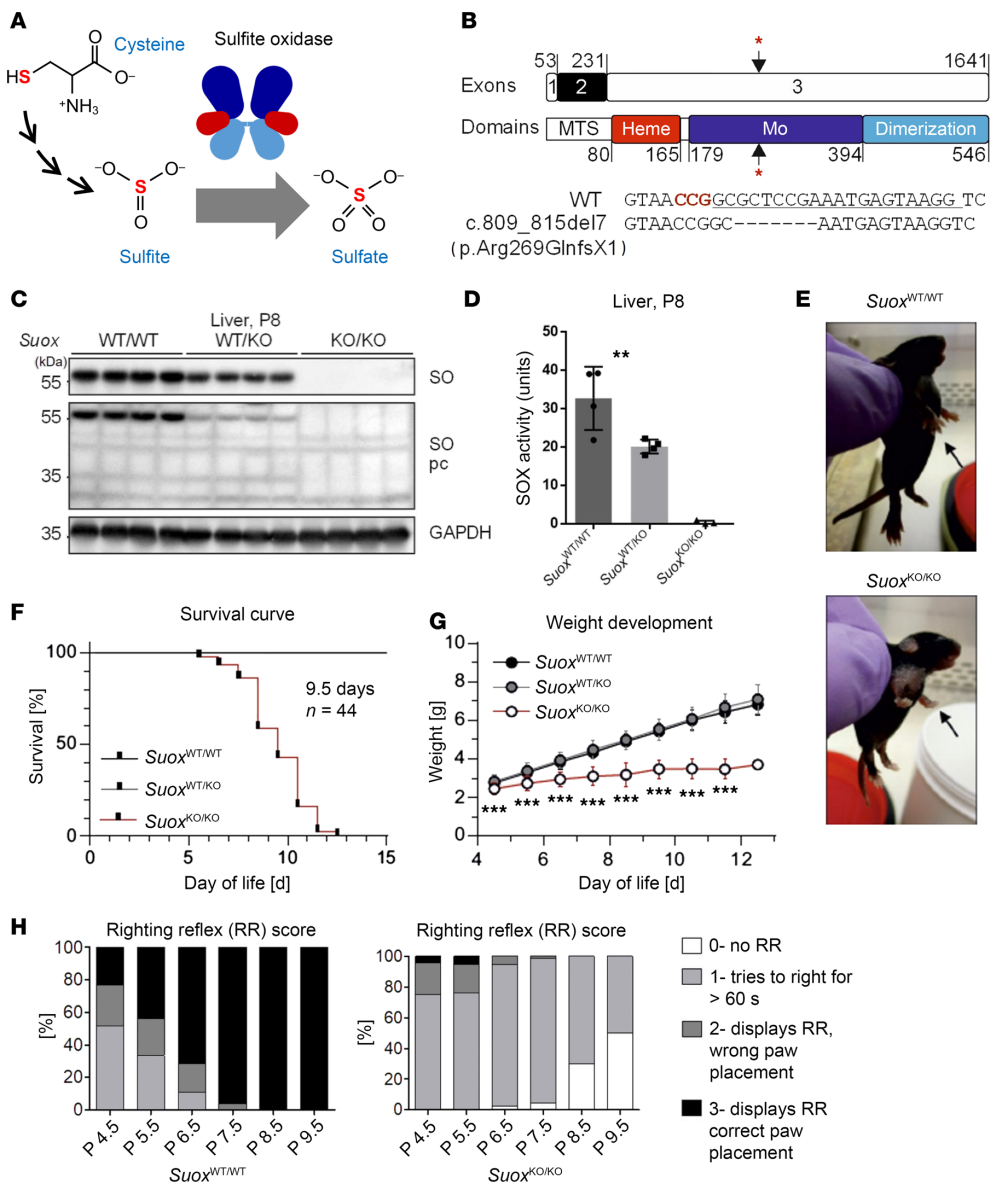
Generation and characterization of *Suox*-KO in C57BL6/J mice. (**A**) Schematic representation of the catalytic function of SOX. (**B**) Schematic representation of murine *Suox* exon structure and SOX domain structure. Asterisks indicate CRISPR/Cas9 targeting site. MTS, mitochondrial targeting sequence. (**C**) Western blot for SOX in liver lysates of littermates from *Suox*^WT/KO^ × *Suox*^WT/KO^ breedings using 2 different antibodies (*n* = 4). (**D**) Cytochrome *c*:sulfite SOX acitivity detected in liver lysates of littermates from *Suox*^WT/KO^ × *Suox*^WT/KO^ breedings (*n* = 8). Student’s *t* test was performed. **P < 0.01; *P < 0.05; NS > 0.05. (**E**) Phenotype of *Suox*^WT/WT^ and *Suox*^KO/KO^ mice at P9. Display of potential front paw spasticity and stunted growth in *Suox*^KO/KO^ mice (bottom) compared with *Suox*^WT/WT^ mice (top). (**F**) Kaplan-Meier plot for survival of *Suox*^KO/KO^ mice (*n* = 28) compared with *Suox*^WT/WT^ (*n* = 25) and *Suox*^WT/KO^ (*n* = 60) littermates. (**G**) Weight development of all genotypes of the SOX-deficient line, starting at P4.5. Asterisks indicate difference of *Suox*^KO/KO^ mice compared with Suox^WT/WT^ and *Suox*^WT/KO^ mice. ***P < 0.001, Student’s *t* test. (**H**) Righting reflex evaluation over time according to criteria modified from Didonato and Bogdanik (61) for *Suox*^WT/WT^ and *Suox*^KO/KO^ mice. Testing was performed at least 3 times per animal and day (technical replicates), with at least 4 animals per genotype and day (biological replicates).

Sulfite oxidase (SOX) deficiencies are life-threatening disorders of sulfur metabolism, impacting the terminal step of cysteine degradation: the oxidation of toxic sulfite to sulfate (Figure 1A). SOX is a molybdenum- and heme-containing dimeric enzyme localized to the intermembrane space of mitochondria, where it transfers electrons derived from sulfite oxidation to cytochrome *c*, thus contributing to mitochondrial respiration. SOX deficiency (SOXD) may either be caused by pathogenic variants in the *SUOX* gene encoding the SOX protein (1, 2), resulting in the so-called isolated SOXD (3), or by defects in the biosynthesis of its molybdenum cofactor (Moco), which is synthesized by a multistep pathway (4) leading to Moco deficiency (MoCD) (5). Both disorders, SOXD and MoCD, are symptomatically similar, underscoring the importance of SOX. Patients with SOXD and MoCD display neonatal feeding difficulties, pharmaco-resistant seizures, severe white matter abnormalities, enlarged ventricles, subcortical cavities, microcephaly, and dysmorphic changes (1, 2, 6, 7). Patients with MoCD also exhibit xanthine urolithiasis due to secondary xanthine oxidase deficiency. The vast majority of all patients exhibit a complete loss of function, and only a minor group represent residual SOX activity causing a delayed onset of the disease with milder symptoms (8).

Sulfite was early recognized to accumulate in urine of patients (9) and later shown to be a reactive, cell-toxic agent (10). We have shown that the accumulating sulfite-cystine adduct *S*-sulfocysteine (SSC) is the key causative factor of neurodegeneration in the disease (11). SSC was found to act as an NMDA receptor agonist, whose effect could be blocked by the application of the NMDA receptor antagonist memantine, inhibiting phenotype development in a pharmacologically induced mouse model of MoCD (11). As a result of SSC accumulation, cysteine/cystine and homocysteine were found to be depleted in SOXD/MoCD patients, suggesting major changes in the overall cysteine homeostasis.

A third accumulating biomarker in SOXD/MoCD is thiosulfate, a catabolic product of sulfur metabolism, which is derived from hydrogen sulfide (H_2_S). H_2_S is synthesized by various enzymes following noncanonical reactions in the trans-sulfuration pathway (cystathionine β-synthase [CBS] and cystathionine γ-lyase [CSE]). In addition, mercaptopyruvate sulfur transferase forms a protein-bound persulfide also contributing to H_2_S formation (12). H_2_S is an important signaling molecule with versatile biological implications. The 2 most well studied mechanisms of its actions are (a) interactions with metalloproteins (13) and (b) redox reactions with thiols in proteins and low molecular compounds (14, 15). H_2_S, cysteine, and thiosulfate metabolic pathways are tightly linked to cysteine persulfidation. This posttranslational modification was reported to have a pivotal role by regulating and protecting functions on thiol residues in proteins (16–18).

Interestingly, thiosulfate also accumulates in ethylmalonic encephalopathy (EE) caused by defects in the enzyme persulfide dioxygenase encoded by the *ETHE1* gene (19). Thiosulfate accumulation in EE is due to accumulating low molecular persulfides formed by sulfide quinone oxidoreductase (SQOR) that are substrates of thiosulfate sulfur transferase forming thiosulfate in a sulfite-dependent manner. EE results in mitochondrial dysfunction, neurodegeneration, and childhood death (20). In addition, SQOR is also able to form thiosulfate in a sulfite- and H_2_S-dependent manner (21). Targeted sulfur metabolome profiling in humans suffering from ultrarare enzyme deficiencies of sulfur metabolic pathways was reported recently, which highlighted that the homeostasis of sulfur metabolites largely relies on catabolic pathways, while H_2_S-synthesizing routes can efficiently compensate for each other’s activity (22).

To date, there are 3 mouse models recapitulating the different forms of MoCD (types A, B, and C) generated by whole-body gene knockouts in *Mocs1*, *Mocs2*, and *gphn* genes (23–25), all presenting with a severe phenotype causing death at the age of 1–8 days. In addition, mice treated with high dosages of tungstate represent a pharmacological model for MoCD (11). All models have in common that in addition to the lack of SOX activity, other Moco-containing enzymes are also dysfunctional. In the case of xanthine oxidoreductase deficiency, accumulating xanthine deposits as kidney stones that may trigger acute kidney failure (25), potentially contributing to the lethality in MoCD mice.

Here, we have generated an animal model for SOXD using CRISPR/Cas9 genome editing to disrupt the murine *Suox* gene. We report the phenotypic characterization and detailed biomarker profile that enabled us to identify a novel contribution of H_2_S biology in the underlying pathomechanism of SOXD. We found a sulfite-dependent liberation of H_2_S from persulfides leading to massive metabolic alterations in SOX-deficient mice.

## Results

### Suox^KO/KO^ mice show a severe phenotype without signs of neurodegeneration.

Using a CRISPR/Cas9 approach, we generated a mouse line with a 7 bp deletion in the *Suox* gene starting at position c.809, causing a frameshift and resulting in a stop codon at position p.Arg269GlnX1 of the SOX protein (Figure 1B). The resulting offsprings segregated as expected in a 1:2:1 manner into *Suox*^WT/WT^, *Suox*^WT/KO^, and *Suox*^KO/KO^, suggesting no lethality during embryo development (Supplemental Figure 1; supplemental material available online with this article; https://doi.org/10.1172/JCI181299DS1). We also observed no sex difference and therefore worked with male and female mice as obtained from breeding. In extracts of liver, the organ with the highest SOX expression (26), we found neither SOX protein nor any detectable SOX activity in *Suox*^KO/KO^, while in *Suox*^WT/KO^, approximately 50% reduced SOX expression was observed (Figure 1, C and D), confirming the successful deletion of the enzyme in *Suox*^KO/KO^ animals.

Phenotypically, *Suox*^KO/KO^ animals displayed delayed fur and whisker development (Figure 1E), similar to *Mocs1*- and *Mocs2*-deficient mice (25). Survival of *Suox*^KO/KO^ mice was significantly impaired, with a lifespan ranging from 5 to 12 days and a median survival of 9.5 days (Figure 1F), which was longer by 1–2 days when compared with *Mocs1*- and *Mocs2*-deficient animals (24, 25), suggesting a minor contribution of the other Moco-dependent enzymes to the pathology of MoCD mice (24, 25). *Suox*^KO/KO^ animals showed postnatal milk spots but failed to thrive starting from P4. Weight gain was significantly impaired following day 4 and was halted or started to decline 1 day before death or reaching a score requiring sacrificing (Figure 1G). Mice used for subsequent analysis were sacrificed at day 8, if not stated otherwise.

Given the neurodegenerative phenotype of human patients (1, 2, 6, 7), we first analyzed mice heads by MRI. *Suox*^KO/KO^ brains appeared round, while the shape of their WT littermates was elliptical, and total brain volume of *Suox*^KO/KO^ mice was decreased by 29.5% when compared with *Suox*^WT/WT^ (Supplemental Figure 2, A and B). Immunohistochemistry for microtubule-associated protein 2 (MAP2) and for the neuronal nuclear antigen (NeuN) revealed no differences between *Suox*^KO/KO^ mice and their WT littermates, as judged by a quantitative analysis in motor cortex layer V (Supplemental Figure 2, C and D). However, when we assessed motor skill development using surface righting reflex testing at P4.5, the scores of the *Suox*^KO/KO^ animals were not strikingly different from *Suox*^WT/WT^ littermates, while motor skill development appeared halted on P5.5 and worsened on subsequent days of testing. At P9.5, approximately 50% of surviving *Suox*^KO/KO^ animals lost any righting reflex, while the remaining animals attempted for longer than 60 s (Figure 1H). In comparison, by P8.5 all WT littermates performed the reflex with correct paw placement (Figure 1H). Inspection of liver and kidney tissue slices representing organs with highest SOX expression did not show any changes in liver (Supplemental Figure 3A), while kidney (Supplemental Figure 3B) showed enhanced focally segmented glomeruli accompanied by minor impairments of filtration function with increased creatinine retention and mild proteinuria (Supplemental Figure 3, B and C). In conclusion, we found that *Suox*^KO/KO^ presented with a severe phenotype, short lifespan and arrest in motor skill development, while no signs of morphological changes in the brain and liver were observed within the first 8 days of life.

### Thiosulfate is the major S-metabolite excreted in Suox^KO/KO^ mice.

**Figure 2:**
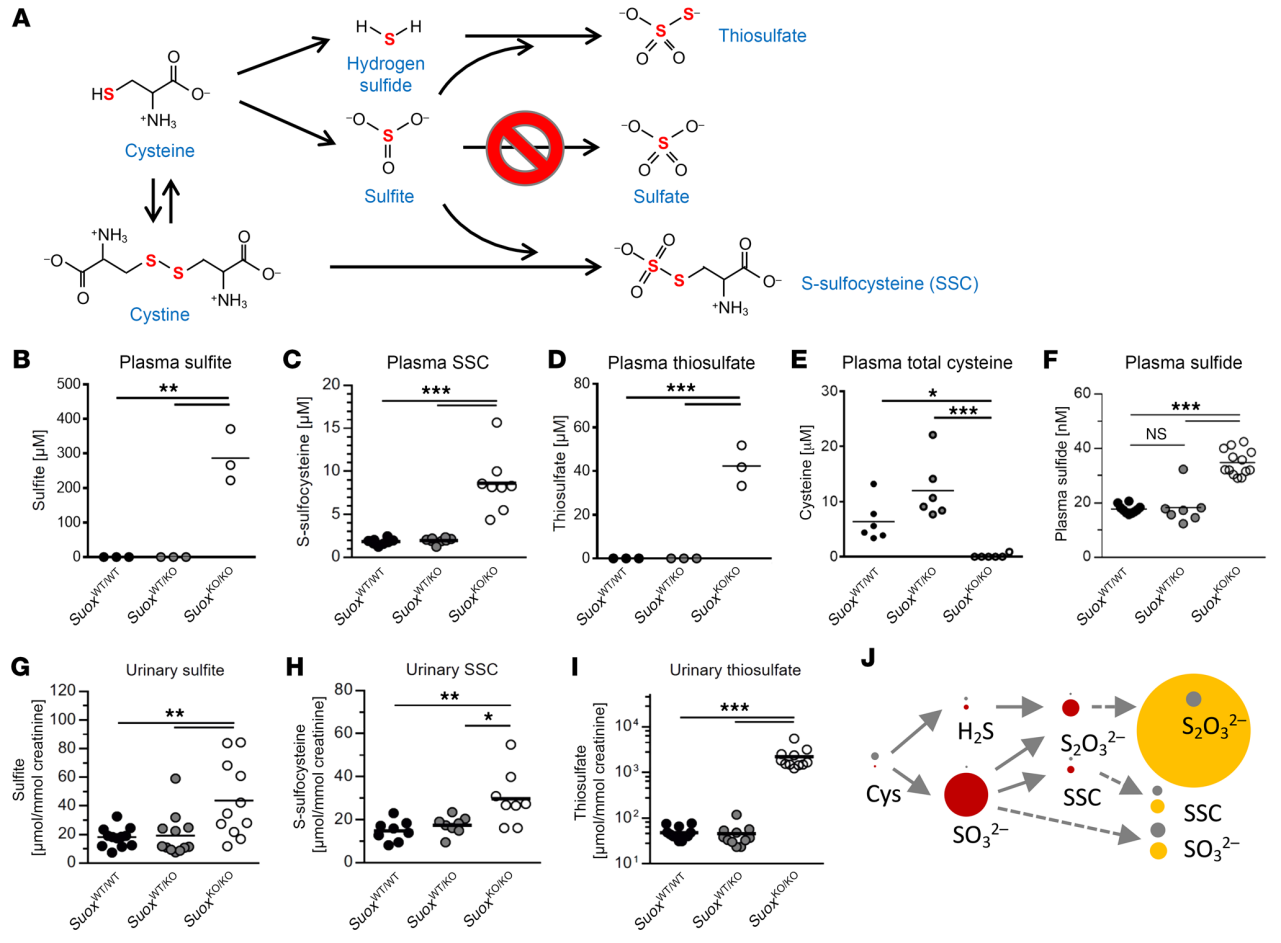
Levels of main biomarkers of SOXD and metabolites of cysteine catabolism in *Suox* mice. (**A**) Schematic representation of cysteine catabolism. Levels of highlighted metabolites (sulfite, SSC, thiosulfate, and H_2_S) and free cysteine determined in this study. (**B**) Determination of plasma sulfite levels in *Suox* mice. (**C**) Determination of plasma SSC levels in *Suox* mice. (**D**) Determination of plasma thiosulfate levels in *Suox* mice. (**E**) Determination of plasma-free cysteine levels in *Suox* strain mice. (**F**) Determination of plasma H_2_S levels in *Suox* mice. (**G**) Determination of urinary sulfite levels in *Suox* mice. (**H**) Determination of urinary SSC levels in *Suox* mice. (**I**) Determination of urinary thiosulfate levels in *Suox* mice. One-way ANOVA with Tukey’s post hoc test for pairwise comparisons was performed. ***P < 0.001; **P < 0.01; *P < 0.05; NS > 0.05. (**J**) Cartoon highlighting the flux of sulfur-containing metabolites in *Suox*^KO/KO^ mice (red circles, plasma; yellow circles, urine) compared with WT mice (gray circles). Circles are drawn in scale to their respective concentrations in plasma and urine, with the exception for H_2_S, which is 10-fold enlarged.

MoCD and SOXD patients display increased urinary sulfite, SSC, *S*-sulfohomocysteine, and thiosulfate (Figure 2A) (22). To understand the flux and organ contribution of these biomarkers, we determined their levels in brain, liver, and kidney as well as in plasma and urine of *Suox*^KO/KO^ mice and control mice (Figure 2, B–D and G–I, and Supplemental Figure 4) and compared these with total cysteine (Figure 2E) and bioavailable H_2_S levels in plasma (Figure 2F).

Sulfite accumulated in all 3 organs, with liver showing the highest sulfite level, as expected for the major SOX-expressing organ (Supplemental Figure 4A). In plasma, sulfite showed the highest level of accumulation, reaching nearly 300 μM (Figure 2B), while urinary sulfite (Figure 2G) was only moderately increased (2-fold), suggesting a poor clearance. For SSC, we found no alteration in liver and kidney and a minor increase in brain (1.6-fold), while in plasma, SSC levels were strongly elevated (4-fold of WT), reaching 8.6 ± 3.4 μM. In contrast, urinary excretion was again only 2-fold increased, when compared with the WT, which is much lower than levels in human patients that show strong accumulation in urine, ranging from 100 to 500 μmol SSC/mmol creatinine (22). Levels of SSC in plasma mirrored the drop in total plasma-free cysteine in *Suox*^KO/KO^ mice, which was not detectable, while WT mice showed 6.3 ± 2.2 μM free cysteine. Based on these data, we conclude that the SSC level reached saturation due to a quantitative conversion of cystine by accumulating sulfite (22, 25).

Thiosulfate, a biomarker of H_2_S catabolism, accumulated in all 3 organs, with the highest levels detected in kidney (Supplemental Figure 4C). Total thiosulfate in plasma reached 42.3 ± 4.4 μM, which was 5 times higher than SSC but much lower than circulating sulfite. To our great surprise, thiosulfate excretion was the highest of all biomarkers, representing 45-fold more than that observed in WT mice. An excretion of 2,200 μmol thiosulfate/mmol creatinine exceeds that of sulfite by almost 40-fold, suggesting that the vast majority of excreted sulfur is derived from H_2_S and related species. Finally, we measured bioavailable H_2_S in plasma (27) and found a significant 2-fold increase in *Suox*^KO/KO^ mice, clearly indicating an alteration in H_2_S metabolic pathways (Figure 2F). Taken together, all biomarker analyses collectively identified a major route for sulfur excretion from accumulating sulfite into thiosulfate (Figure 2J).

Given the metabolic link between thiosulfate and H_2_S, we next investigated H_2_S biogenesis, homeostasis, and catabolism in *Suox*^KO/KO^ mice. In a proteome-wide analysis (Supplemental Figure 4), we inspected levels of enzymes involved in H_2_S biogenesis and found no biologically relevant alteration for CBS, CSE, and 3-mercaptopyruvate sulfurtransferase in liver and kidney, thus excluding increased H_2_S biosynthesis as a source of H_2_S and thiosulfate (Supplemental Figure 5A). SQOR, persulfide dioxygenase, and thiosulfate sulfurtransferase (TST) catalyze individual steps in the H_2_S oxidative pathway, producing glutathione and other low–molecular weight persulfides that are further converted into sulfite and thiosulfate (28, 29). To further inspect the expression level of these H_2_S-metabolizing enzymes, we performed Western blot analyses to detect SQOR and TST expression in liver and kidney (Supplemental Figure 5B) as well as in tungstate-treated HEK293 cells and patient fibroblasts (Supplemental Figure 5, C and D). We found a significant reduction in SQR expression (except in liver) and a minor reduction of TST in patient cells; neither finding explained the massively increased thiosulfate excretion.

Out of all the proteins involved in cysteine metabolism, there was only 1 enzyme showing a significant change in our proteomic study (Supplemental Figure 5A): cysteine dioxygenase 1, the first enzyme in oxidative cysteine catabolism, was 7.3-fold reduced, which is in line with the loss of free cysteine in plasma (30). Therefore, the observed increase in H_2_S and the massive excretion of thiosulfate should have a different origin, which we investigated next.

### Sulfite induces a release of H_2_S from persulfides.

**Figure 3:**
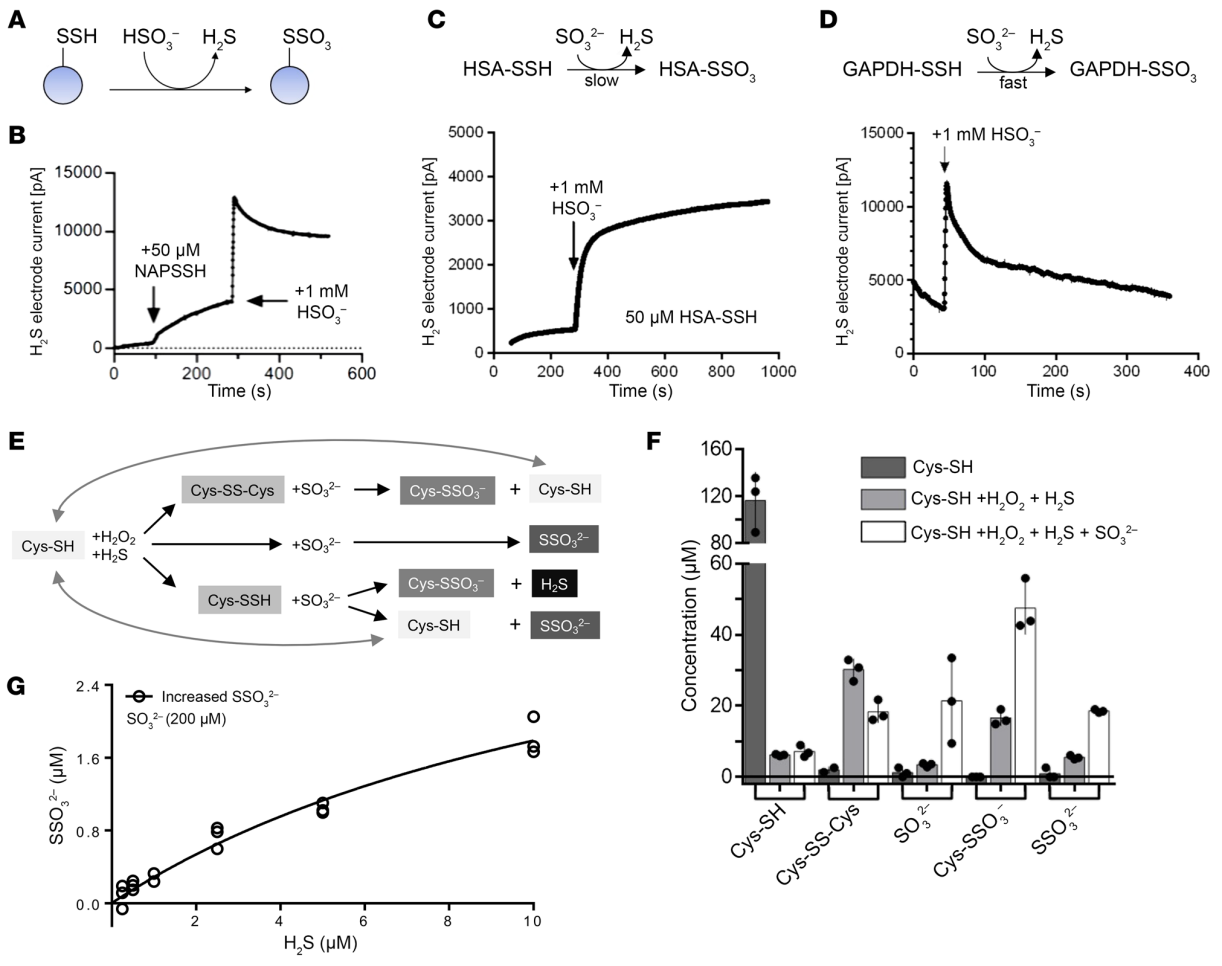
Sulfite triggers protein depersulfidation and releases H_2_S. (**A**) Schematic reaction of persulfides with HSO_3_^–^ at pH 7.4, producing persulfonates (*S*-sulfonates) and releasing H_2_S. (**B**) Representative reaction of 50 μM NAP-SSH with 1 mM HSO_3_^–^ at pH 7.4, measuring H_2_S release. (**C**) H_2_S electrode signal of 50 μM HSA-SSH in 50 mM PBS, pH 7.4, reacting with a 20-fold molar excess of sulfite (*n* = 3). (**D**) H_2_S electrode signal of 50 μM GAPDH-SSH in 50 mM PBS, pH 7.4, reacting with 20-fold molar excess of sulfite (*n* = 3). (**E**) Schematic representation of cysteine persulfides depersulfidated by sulfite and formed H_2_S. (**F**) End-product measurements of in vitro interaction between sulfite and cysteine persulfides. Error bars indicate SD. (**G**) H_2_S-dependent nonenzymatic thiosulfate formation with a high level of sulfite.

In light of the accumulation of sulfite observed in all investigated organs, with the highest concentration in plasma, we raised the question whether sulfite reacts with persulfidated small molecules and proteins known to contribute significantly to the overall pool of labile H_2_S in the organism (18, 31). We hypothesized that sulfite treatment of persulfides could result in the formation of S-sulfonylated species with the concomitant release of H_2_S (Figure 3A) (18). First, we tested the reactivity of sulfite toward persulfides using an H_2_S electrode and various low–molecular weight and protein persulfide models. *N*-acetylpenicillamine persulfide (NAP-SSH) was reported to display slow H_2_S dissociation kinetics at pH 7.4 (32). When adding of 20-fold molar excess of sulfite (1 mM) to 50 μM NAP-SSH, the dissociation rate of H_2_S was greatly increased within seconds, leading to a rapid reequilibration (Figure 3B). Next, we investigated 2 different protein model substrates previously reported to be persulfidated at key cysteine residues (33): HSA (Figure 3C) and GAPDH (Figure 3D) (34–36). Similar to NAP-SSH, sulfite-dependent H_2_S release from GAPDH was observed with a rapid H_2_S spike and reequilibration (Figure 3D), collectively confirming a nonenzymatic release of H_2_S from various persulfidated targets.

The reaction of sulfite with persulfides also displays a path toward S-sulfonylation other than the oxidation of persulfides (17, 18). To monitor this half of the reaction and to answer the question as to which extent sulfite reacts with the outer-sphere sulfur of the persulfide leading to thiosulfate and free thiol release, we designed another in vitro experiment (Figure 3E). As described before, mild oxidation of cysteine to sulfenic acids increased the reactivity of thiols toward H_2_S, thus forming cysteine persulfides in vitro (18).

We treated 100 μM cysteine with equimolar amounts of H_2_O_2_ and H_2_S for 1 h and determined the amount of cysteine, cystine, sulfite, SSC, and thiosulfate (Figure 3F). As expected, we found 30 μM cystine, representing 60% of cysteine being oxidized by H_2_O_2_. However, approximately 18 μM SSC and 7 μM thiosulfate were also formed, leaving only approximately 15% of the remaining cysteine to be potentially persulfidated. When treating this mixture with 100 μM sulfite, approximately 10 μM cystine was depleted, resulting in the formation of SSC and cysteine. The fact that 30 μM additional SSC was formed suggests that cysteine-persulfide donated a significant fraction (~10 μM) to this reaction. The fact that only 20 μM remaining sulfite was detected suggests that a large fraction of sulfite was bound in SSC. In addition, we found an increase in thiosulfate formation that could either suggest an abstraction of the persulfide’s outer sulfur by sulfite and/or an H_2_S-dependent formation of thiosulfate with sulfite. We have shown the existence of the latter reaction by titrating sulfite with substoichiometric amounts of H_2_S leading to a thiosulfate/H_2_S ratio of approximately 1:4 in the presence of sulfite excess (200 μM; Figure 3G), conditions representing levels in *Suox*^KO/KO^ mice. In aggregate, our in vitro studies demonstrate that at pathological sulfite concentrations, various persulfides release H_2_S, resulting in the formation of S-sulfonylated reaction products.

### Sulfite changes the persulfidome of Suox^KO/KO^ mice.

**Figure 4:**
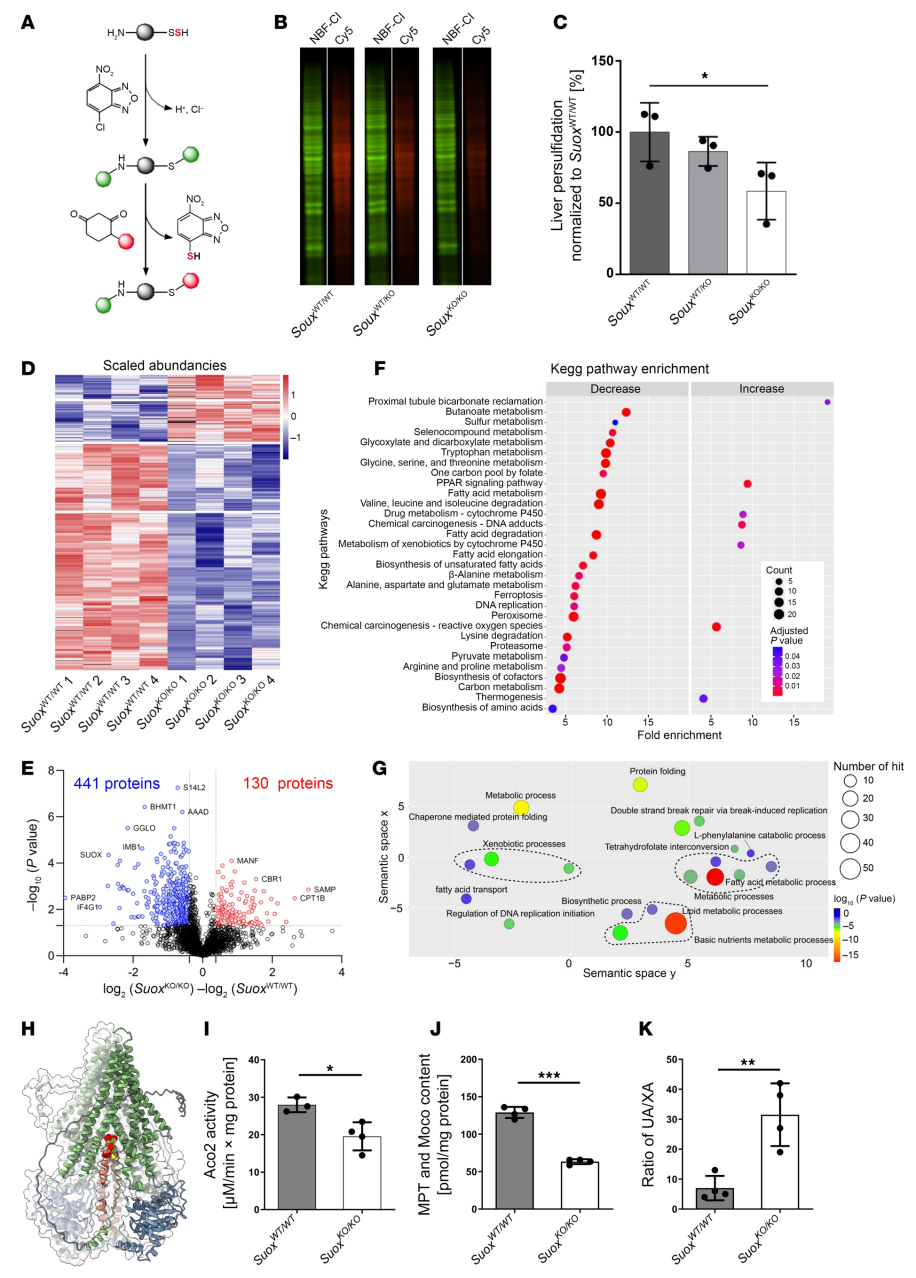
Persulfidome remodeling in *Suox*^KO/KO^ mice. (**A**) Setup for the detection of protein-bound persulfides in the assay described by Zivanovic et al. (18). Crude extract from liver was treated with 4-chloro-7-nitrobenzofurazan (NBF-Cl), which reacts with persulfides, free thiols, sulfenic acids, and amine groups. The NBF-blocked persulfides undergo nucleophilic attack by the DAz-2 moiety, which in turn is cross-linked to a fluorescent Cy5 moiety via click chemistry. (**B**) Representative Cy5 (persulfonate) and NBF-Cl (control) fluorescence signals measured by a fluorescence scanner (Cy5, red; NBF-Cl, green). (**C**) Quantification of a specific rectangular area identical for all lanes. All Cy5 signals were normalized to their respective NBF-Cl signals and further normalized to the mean of *Suox*^WT/WT^ signals (*n* = 3). Error bars indicate SD. One-way ANOVA was performed. (**D**) Heatmap showing the significant changes of protein persulfidation in *Suox*^KO/KO^ mouse livers compared with WT animals (Welch’s test, *P* < 0.05). (**E**) Volcano plot depicting statistical significance plotted against the log_2_ fold change of persulfidated proteins in *Suox*^KO/KO^ mouse liver relative to WT animals. Significance was established using Welch’s *t* test (2 sided), with a *P* value threshold of <0.05. Fold change cutoffs were established at 30%. The numbers of proteins with significantly altered persulfidation states are indicated. (**F**) KEGG pathway enrichment analysis using DAVID. The graph shows the top 30 significant (Benjamini’s adjusted *P* value < 0.01) and most enriched terms, with color gradient signifying the adjusted *P* value and circle size the number of proteins. (**G**) GO (Biological Process) term enrichment analysis of the 441 proteins found to have decreased persulfidation levels in *Suox*^KO/KO^ mice. REVIGO was used to plot the enrichment analysis performed in DAVID. Circle dimensions denote the protein count within specific GO terms, while color gradients depict the degree of significance. Similar biological processes are grouped together with a dashed line. (**H**) AlphaFold model of human ABCB7 protein with the C-terminal helix pointing into the transport pore. The C-terminal Cys residues are highlighted in a spheres model. (**I**) Determination of cytosolic Aco2 activity, (**J**) molybdopterin (MPT)/Moco content via HPLC Form A analysis, and (**K**) urinary uric acid/xanthine (UA/XA) ratio. ***P < 0.001; **P < 0.01; *P < 0.05.

Following our findings that sulfite reacts with persulfides, we wanted to test how accumulation of sulfite affects the global persulfidome landscape. Using the dimedone switch method (18) for selective persulfide labeling (Figure 4A), we first observed, in gel, that liver samples from *Suox*^KO/KO^ mice show significantly lower protein persulfide levels (Figure 4, B and C). To quantify this change and understand its biological effect, we next performed a label-free persulfidome analysis (37) and identified 2,457 persulfidated proteins in both WT and *Suox*^KO/KO^ mouse liver samples (Supplemental Table 1). Among these, 571 proteins passed the significance threshold (presence in 3 out 4 samples, at least 30% change and *P* < 0.05), of which 441 proteins showed a clear decrease in protein persulfidation (Figure 4, D and E, and Supplemental Figure 6, A and B). To exclude that this change originates from the change in total protein level, we also performed label-free total proteome analysis (Supplemental Figure 6, C and D) and double plotted the persulfidome change to the total proteome change (Supplemental Figure 6E). Indeed, a majority of identified proteins seems to be unaffected by the change in protein expression levels.

Kyoto Encyclopedia of Genes and Genomes (KEGG) pathway enrichment analysis of proteins that showed significant decrease in protein persulfidation suggested that a variety of metabolic processes are affected, such as amino acid metabolic pathways, lipid metabolism, and pyruvate metabolism, but also ferroptosis, DNA replication, and PPAR signaling, etc. (Figure 4F). A complementary Gene Ontology (GO) term enrichment analysis identified similar pathways (Figure 4G). Finally, the loss in S-persulfidation in the proteome was also accompanied by an increase S-sulfonylation (Supplemental Figure 7).

When inspecting the list of depersulfidated proteins, the mitochondrial Fe-S cluster transporter ABCB7 caught our attention. ABCB7 is involved in the export of an Fe-S cluster species to the cytosol, serving as precursor for the cytosolic Fe-S cluster biogenesis (38), and levels of ABCB7 persulfidation were decreased 5-fold (rank 13). Recent structural studies provided evidence that glutathione-complexed [2Fe-2S] clusters serve as substrates of the transporter (39). Mouse and human ABCB7 harbor 6 cysteine residues, 3 of which are located at the C-terminal tail of the transporter for which structural information is missing as of today. Modeling the structure of mouse ABCB7 using AlphaFold predicted the C-terminal helix to be localized within the transport pocket, with its terminal 3 cysteines pointing to the center of the complex (Figure 4H). Therefore, we probed the impact of sulfite-induced depersulfidation on cytosolic Fe-S clusters. Cytosolic aconitase activity was reduced by approximately 30% (Figure 4I). Total Moco/molybdopterin content showed a more than 50% reduction (Figure 4J), which was consistent with earlier studies using ATM1/ABCB7-deficient plants (40). Finally, we determined levels of xanthine and uric acid, substrates and products of xanthine oxidase, a cytosolic Moco and Fe-S cluster–dependent enzyme. Again, we found a highly significant and strongly increased ratio of uric acid to xanthine, suggesting a reduction of xanthine oxidase activity in our SOX-deficient mouse (Figure 4K). Therefore, we conclude that sulfite-induced depersulfidation compromises cytosolic Fe-S cluster biogenesis, thus underlining the pleiotropic effect of accumulating sulfite in various downstream pathways.

### Changes in the amino acid profile suggest metabolic rewiring.

We have conclusively demonstrated that sulfite toxicity in mice resulted in H_2_S release from S-persulfidated stores, leading to a massive excretion of thiosulfate. H_2_S toxicity has been attributed to the inhibition of mitochondrial respiration due to the blockage in complex IV (41). A recent study by Banerjee and coworkers showed that H_2_S excess may cause reversal of the respiratory chain and an increase in mitochondrial NADH pool, thus leading to a partial reversal of the TCA cycle with increased glycolysis to generate the required ATP (42). To feed the TCA cycle, increased glutaminolysis acts as the major source for α-ketoglutarate (42).

**Figure 5:**
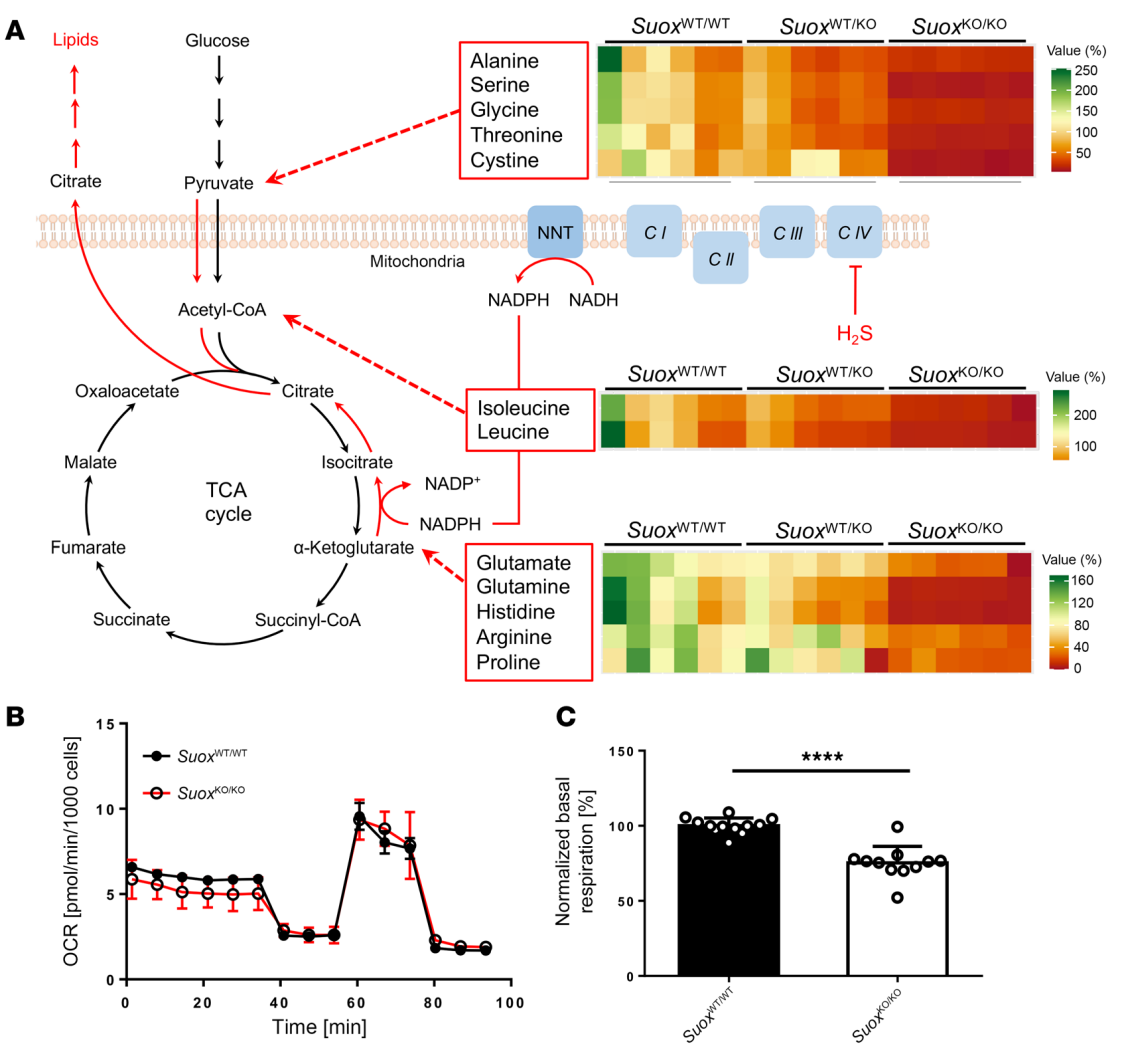
Metabolic impacts of elevated H_2_S in *Suox*^KO/KO^ mice. (**A**) Schematic representation of partial reversal of TCA cycle and relative changes (*n* = 6) of urinary amino acids compared with *Suox*^WT/WT^. Amino acids are grouped according to their contribution as catabolic precursors of α-ketoglutarate, acetyl-CoA, and pyruvate. (**B** and **C**) Mitochondrial respiration of mouse embryonic fibroblasts from *Suox*^WT/WT^ and *Suox*^KO/KO^ mice showing a representative trace (**B**) and normalized basal oxygen consumption rate (OCR) (**C**). Two-tailed unpaired Student’s *t* test was used to compare 2 groups. Data are presented as mean ± SEM. ****P < 0.0001.

To obtain further insights into the metabolic changes in *Suox*^KO/KO^ mice, we determined the amino acid profile in urine (Figure 5A) and plasma (Supplemental Figure 8). Remarkably, levels of nearly all amino acids were decreased in *Suox*^KO/KO^ mice, with cystine and glutamine showing the strongest reduction (Figure 5A). While cystine depletion is directly related to quantitative conversion to SSC, an over 10-fold reduction in glutamine supports the above-mentioned consequences of H_2_S toxicity on mitochondrial respiration. In addition, all other catabolic amino acid precursors (Glu, His, Arg, and Pro) of α-ketoglutarate were also reduced (Figure 5A). Furthermore, amino acids contributing to acetyl-CoA and pyruvate formation (Ala, Ser, Gly, Thr, and Cys) were also depleted, collectively arguing for a metabolic arrest in *Suox*^KO/KO^ mice. This is in line with the persulfidome data and the observation that animals stop gaining weight at day 4, suggesting a catabolic state of metabolism.

We also investigated embryonic fibroblasts generated from our *Suox*^KO/KO^ mice and performed mitochondrial respiration studies (Figure 5B). Basal respiration was significantly compromised in *Suox*^KO/KO^ cells (Figure 5, B and C), which is consistent with a sulfite-dependent release of H_2_S, thus blocking complex IV.

### Therapeutic sulfite-scavenging approach.

**Figure 6:**
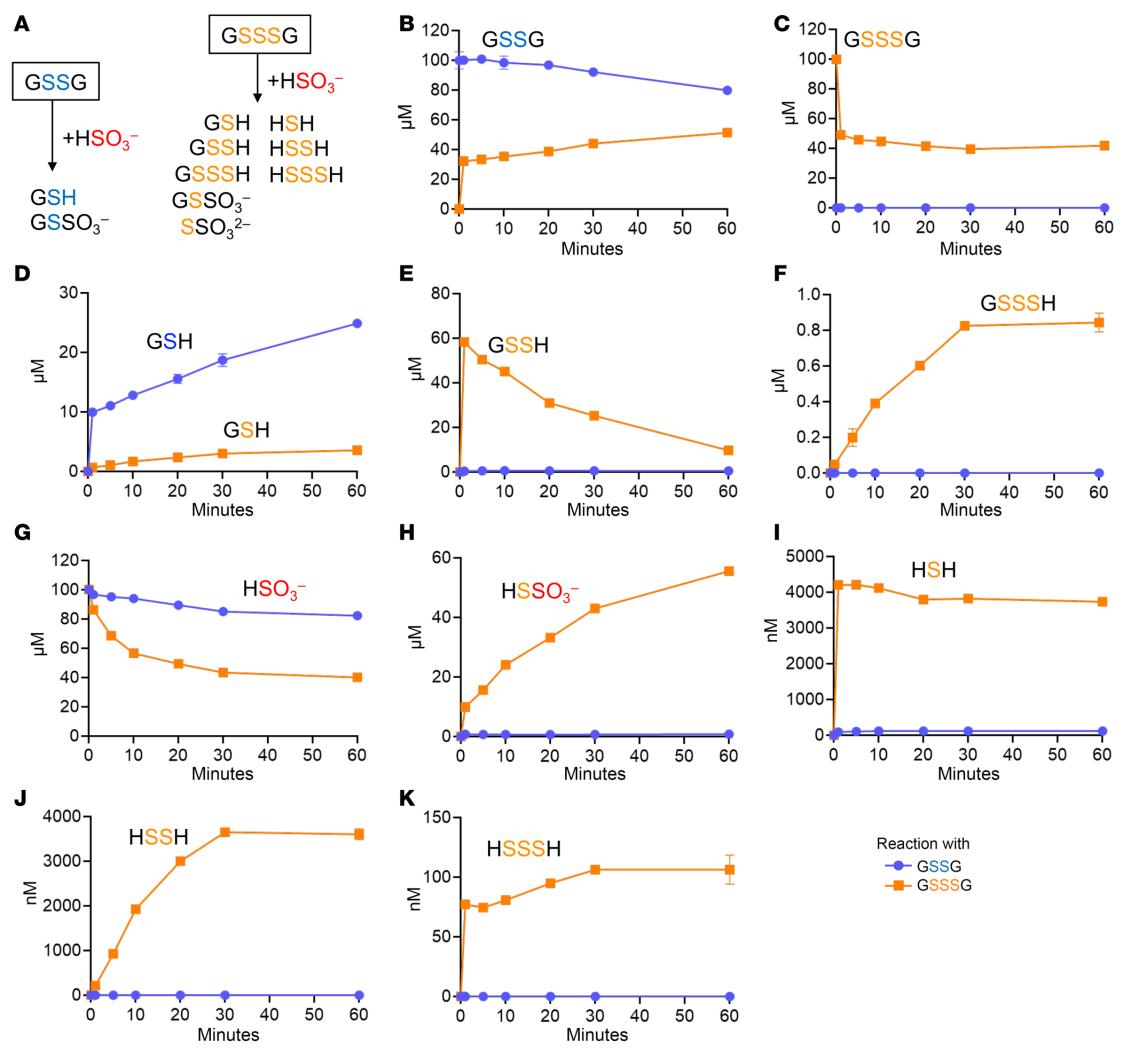
In vitro reaction of sulfite with GSSG and GSSSG. (**A**) Expected species formed by the reaction with sulfite. (**B**–**F**) Determination of glutathione products and (**G**–**K**) sulfite and S-related products using mass spectrometry following 1 h of incubation. The kinetics of the reaction are shown in Supplemental Figure 8.

After showing that sulfite reacts with persulfides, we wanted to probe whether externally applied disulfide and polysulfides could efficiently scavenge accumulating sulfite in *Suox*^KO/KO^ mice. Therefore, we used oxidized glutathione (GSSG), which is known to be excreted from cells (43, 44). In addition, we used glutathione trisulfide (GSSSG), which produces GSSH upon reaction with endogenous GSH (45, 46). We first tested the in vitro reactivity of 100 μM sulfite incubated with equimolar amounts of GSSG and GSSSG for up to 60 min (Figure 6 and Supplemental Figure 8) and expected the formation of GSH, sulfoxidated GSSO_3_^–^, and various persulfidated species and hydrosulfides (Figure 6A). While GSSG was only partially depleted (Figure 6B), GSSSG was rapidly consumed in a sulfite-dependent manner, thus suggesting a much higher reactivity with sulfite (Figure 6C and Supplemental Figure 8). In parallel, GSH accumulated over time in the reaction of GSSG, while it accumulated much less with GSSSG (Figure 6D), with the initial fast formation of GSSH (Figure 6E) and slow accumulation of GSSSH (Figure 6F). Consistently, more sulfite was depleted in the presence of GSSSG (Figure 6G), which was accompanied by the formation of thiosulfate (Figure 6H and Supplemental Figure 9), thus suggesting a sulfite-dependent release from the reactive persulfide intermediate GSSH. Finally, H_2_S and hydropolysulfides were also formed following the reaction of GSSSG with sulfite at low micromolar (H_2_S and H_2_S_2_) to nanomolar levels (H_2_S_3_; Figure 6, I–K). In aggregate, when summing up reactants consumed and products formed, we conclude a quantitative interconversion of GSSSG into GSSG and thiosulfate (Supplemental Figure 9, K–M).

**Figure 7:**
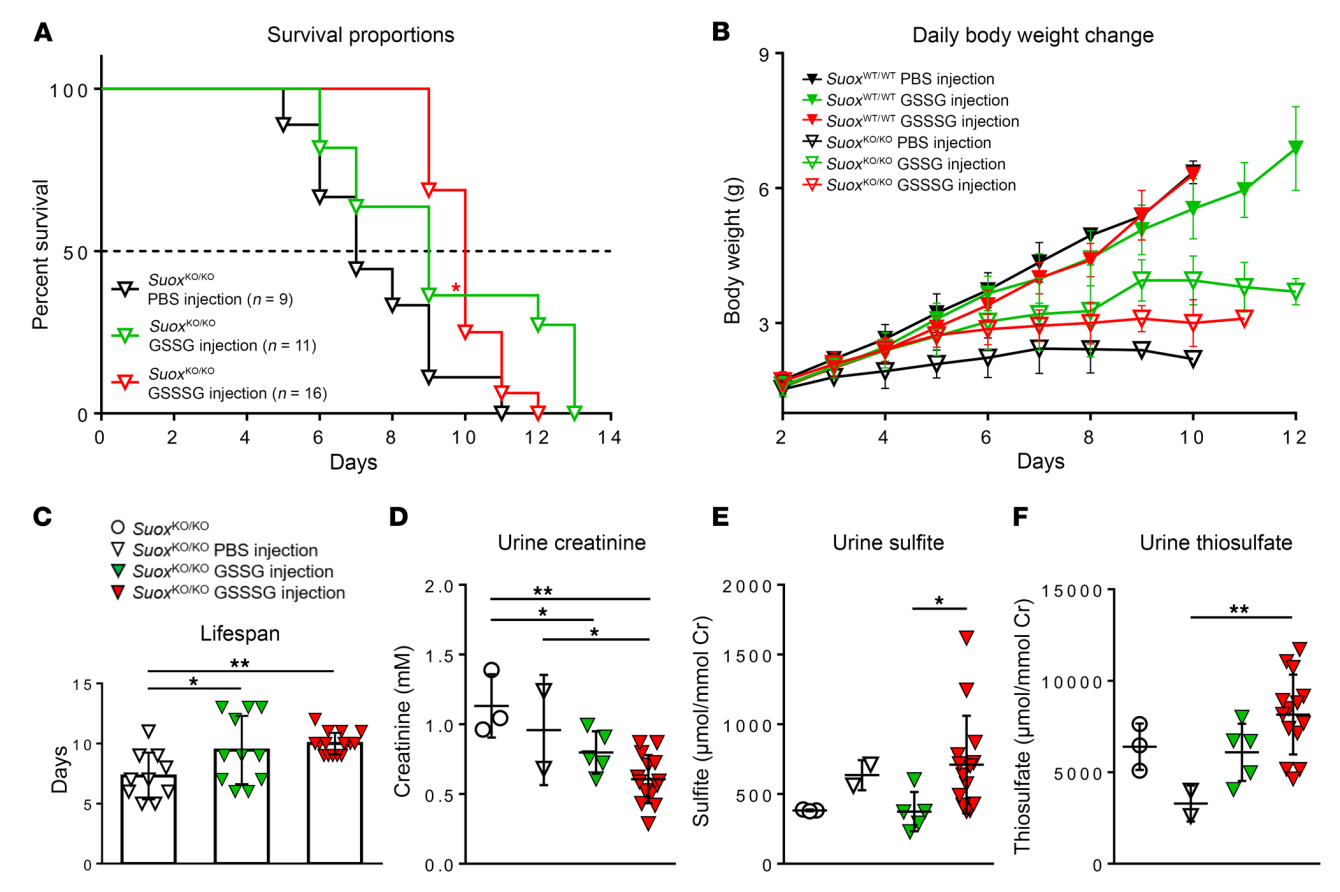
Treatment of WT and *Suox*^KO/KO^ mice with GSSG and GSSSG. (**A**) Kaplan-Meier survival curves were compared using 2-sided log-rank (Mantel-Cox) tests for 2 prespecified pairwise comparisons; unadjusted *P* values are reported. *P < 0.05. (**B**) Body weight development for animals treated intraperitoneally with 2 mM GSSG and 2 mM GSSSG. (**C**) Comparison of lifespan between PBS- and GSSG/GSSSG-treated *Suox*^KO/KO^ mice. (**D**–**F**) Urinary biomarker analyses of creatinine (**D**), sulfite (**E**), and thiosulfate (**F**). (**C**–**F**) One-way ANOVA was performed, with Tukey’s post hoc test for pairwise comparisons; Tukey-adjusted 2-sided *P* values are reported. ***P < 0.001, **P < 0.01, *P < 0.05.

Next, we treated newborn mice derived from *Suox*^KO/WT^ breeding with either PBS or PBS containing 2 mM GSSG or GSSSG intraperitoneally. The amount was calculated based on the sulfite concentration determined in *Suox*^KO/KO^ mice (~250 μM), aiming to neutralize sulfite excess with the applied sulfite-scavenging glutathione species. We observed an extension in lifespan of *Suox*^KO/KO^ mice treated either with GSSG or GSSSG by 2 and 3 days, respectively (Figure 7, A and C). The extension in lifespan was highly significant for GSSSG-treated animals, while the improved body weight gain was higher for animals treated with GSSG (Figure 7B). WT animals showed no alteration in survival (data not shown) or growth (Figure 7, B and C). Biomarker analysis showed that kidney function improved in both cohorts (Figure 7D), while sulfite and thiosulfate accumulation did not change under GSSG treatment or even further increased following GSSSG exposure (Figure 7, E and F). Although both sulfite-scavenging molecules were able to extend lifespan, the additional intake of glutathione as a cysteine-containing metabolite further exaggerated cysteine catabolism, leading to even more sulfite intoxication. The fact that lifespan was extended suggests a minor delay in reaching the pathological concentration of sulfite and underlines the high reactivity of sulfite toward oxidized and persulfidated thiols.

Taken together, SOX-deficient mice displayed an unexpected rewiring of cysteine catabolism into the H_2_S pathway caused by a sulfite-induced depersulfidation, H_2_S release, and thiosulfate accumulation, which together with our pilot treatment studies suggests that sulfite scavenging may provide a new route for treating this ultrarare and devastating metabolic disorder.

## Discussion

Based on the symptomatic similarity between MoCD and SOXD, defective SOX enzyme is recognized as the major cause of death in both inborn errors of metabolism causing early childhood death. So far, only MoCD mice models were described; thus, to our knowledge, we generated and characterized the first SOXD mouse model. Human patients with SOXD were reported with feeding difficulties and unprovoked and drug-resistant seizures shortly after birth, and they further developed secondary pathologies such as lens dislocation, neurodegeneration with cyst formation, and microcephaly leading to a mean survival of 4 years (47). Remarkably, MoCD/SOXD patients are often misdiagnosed with hypoxic ischemia encephalopathy (48). In comparison, *Suox*^KO/KO^ mice had severe growth retardation — they stopped gaining weight from P4 — that resulted in a mean lifespan of 9.6 days.

Although *Suox*^KO/KO^ mice die at a young age, neurodegeneration was not found in them. In addition, in previously reported MoCD mouse models, including both *Mocs*1- (24) and *Mocs*2-deficient (25) mice, no sign of neurodegeneration were reported. This may have occurred for the following reasons: (a) Brain development in newborn rodents is not as complete as in human infants. Rodent brains at P7–P10 are equivalent to human infant brains (49). Given that *Suox*^KO/KO^ mice die within 9.6 days, we expect to be outside the developmental window of the comparable age of human patients. (b) Urinary SSC levels in mice were at 30 μmol/mmol creatinine, which was much lower than in human patients (129 μmol/mmol creatinine) (22), suggesting a reduced overall production and clearance rate of SSC. Given that plasma cysteine was completely depleted in *Suox*^KO/KO^ mice, we conclude that limiting cystine concentrations could be the primary cause of lower SSC levels in mice. Therefore, we considered other alterations in sulfur-containing metabolites as drivers for the disease pathology in mice.

When comparing the excretion of sulfite (45 mmol/mol creatinine), SSC (32 mmol/mol creatinine), and thiosulfate (2,200 mmol/mol creatinine), it became clear that the vast majority (97%) of sulfur is excreted as thiosulfate, the catabolic oxidation product of H_2_S and sulfite. Thus, we wondered if H_2_S and thiosulfate also contributed to the SOXD pathomechanism. Patients with 2 other inherited metabolic disorders, SQOR deficiency (SQORD) and EE, were also reported to have approximately 2-fold increased levels of H_2_S, but only EE patients showed thiosulfate elevation comparable with MoCD/SOXD patients in plasma (22). SQORD is caused by the mutations in the *SQOR* gene, encoding for a mitochondrial enzyme that oxidizes H_2_S to persulfides (50, 51). SQOR is also able to form thiosulfate in vitro in a sulfite- and H_2_S-dependent manner (21). Given that SQOR expression was reduced in liver, kidney, and various SOX-deficient cells, we assumed that SQOR was not the major driver of thiosulfate formation in *Suox*^KO/KO^ mice (Supplemental Figure 5). However, EE patients (52) accumulated persulfides, which are further metabolized in a sulfite-dependent manner by TST, yielding thiosulfate. Remarkably, similar to MoCD and SOXD, patients with SQORD or EE suffer from brain lesions, which are considered to be the consequence of H_2_S accumulation.

H_2_S is gaining growing interest as a gasotransmitter due to its role in mediating persulfidation of proteins and small molecules with beneficial effects on stress resistance and longevity (18). However, at high concentrations, H_2_S binds to cytochrome *c* oxidase, thus suppressing mitochondrial oxidative phosphorylation, as shown in fibroblasts derived from SQORD and EE patients (52, 53). In contrast to our SOXD mouse model, SQORD and EE mice (41, 54) showed milder disease progression with average lifespan of weeks to months at comparable levels of H_2_S. *Sqor*^–/–^ mice were indistinguishable from WT littermates before weaning, but later developed ataxia resulting in premature death at the age of 10 weeks. By contrast, *Ethe1*^–/–^ mice had a more severe phenotype than *Sqor*^–/–^ mice, given that they showed growth arrest from P15 and died between 5 and 6 weeks of age. In addition, a large and comparative study that included several sulfur metabolic enzyme disorders in humans showed that in addition to MoCD/SOXD patients, EE patients also showed moderate but significant accumulation of sulfite (22). In aggregate, our findings suggest that SOXD resulted in a massive accumulation of both sulfite and thiosulfate in mice, leading to a collective pathology of sulfite and H_2_S metabolites, given that thiosulfate has been shown to be rather safe (11) and even protective (55, 56).

The observed large increase in plasma H_2_S in *Suox*^KO/KO^ mice is comparable with that seen in EE patients (22); however, none of the enzymes involved in H_2_S biosynthesis or catabolism were found to be significantly altered, except for a mild reduction in SQOR. Therefore, we hypothesized that other sources of H_2_S-derived thiosulfate may exist. Sulfite attacks various forms of oxidized cysteines, as known for cystine-dependent SSC formation (11), and we demonstrated here that sulfite also cleaves the S-S bond of persulfidated cysteines, either in the metabolite cysteine-persulfide or in peptides and proteins. The chemical properties of persulfides are unique in the sense that the inner and outer sulfurs are not equivalent in their reactivity. In comparison, the pK_a_ value of RSH is approximately 8–10, while the pK_a_ value of H_2_S is approximately 7, which makes HS^–^ a better leaving group than RS^–^ (57). To study which sulfur atom is the main site for the nucleophilic attack of sulfite and which reaction products are formed, we treated persulfidated NAP, HSA, and GAPDH with sulfite and observed different kinetics for H_2_S release. When using in vitro–generated Cys-SSH, sulfite treatment resulted in a near stoichiometric formation of SSC. In addition, a minor formation of thiosulfate was observed due to H_2_S- and H_2_O_2_-dependent reactions with sulfite. Therefore, we conclude that sulfite-induced release of H_2_S from persulfidated stores leads to an acute H_2_S intoxication and loss of protein persulfidation. Consistently, our proteome-wide study in liver extracts of *Suox*^KO/KO^ mice identified a significant and widespread change in protein persulfidation of more than 400 proteins, most of which were involved in metabolic pathways. Furthermore, consistent with our in vitro studies using Cys-SSH, we found an increase in S-sulfoxidated peptides in liver extracts of *Suox*^KO/KO^ mice.

Protein persulfidation protects the respective proteins from sulfonylation (R-SO_3_H) of cysteine residues and a subsequent loss of protein functions. Currently, the effect of reduced protein persulfidation has been mainly studied in the context of aging (18), and its impacts on the specific proteins remain sparse, with only a handful of examples, such as the persulfidation on human GAPDH (Cys152), human CSE (Cys252, 255, 307, and 310), and human eNOS (Cys443), which increase the respective enzymes’ activities, while the persulfidation on human PTP1B (Cys215) suppresses its activity (58, 59). We chose one of the top-ranked depersulfidated proteins, ABCB7, the Fe-S cluster precursor transporter in the inner mitochondrial membrane, and demonstrated by 3 independent biochemical analyses that its loss in persulfidation correlated with reduced cytosolic Fe-S cluster biogenesis. In summary, our study identified a large pool of proteins with sulfite-sensitive persulfides that may collectively or individually have contributed to the disease pathology in SOXD. Besides a better understanding of SOXD, we have also identified crucial persulfidation sites in the proteome that may contribute to stress resistance and longevity.

Sulfite-induced release of H_2_S from persulfides is considered a transient process with a yet unknown concentration of H_2_S at peak reaction. Given the clinical similarity of MoCD/SOXD with hypoxic ischemia encephalopathy, we speculate that the proposed H_2_S peak would cause a transient inhibition of cytochrome *c* oxidase and mimic hypoxic conditions. In contrast, at moderately increased H_2_S levels, a recent study demonstrated increased mitochondrial respiration due to efficient H_2_S oxidation by SQOR (21). When a critical concentration of H_2_S is reached, cytochrome *c* oxidase is inhibited, resulting in respiration arrest, increased NADH levels, and reversal of the TCA cycle, as demonstrated by glutaminolysis, to replenish α-ketoglutarate (42). By investigating the amino acid profile in *Suox*^KO/KO^ mice, we found a depletion of all catabolic amino acid precursors of α-ketoglutarate, with the highest reduction in glutamine levels, thus providing in vivo support for a H_2_S-dependent reversal of the TCA cycle. Consistently, basal mitochondrial respiration was reduced in *Suox*^KO/KO^ MEFs. Finally, we demonstrated the critical importance of sulfite scavenging and its reactivity toward persulfides for the treatment of *Suox*^KO/KO^ mice. While our in vitro experiments showed a preferred reaction of sulfite with GSSSG over GSSG, both treatments resulted in a moderate extension in lifespan by 2–3 days. The concomitant increase in the disease biomarkers sulfite and thiosulfate is due to the additional intake of sulfur by applying glutathione species and underlines the necessity to develop sulfur-free forms of sulfite scavengers for future therapeutic approaches.

## Methods

### Sex as a biological variable

Both male and female mice were analyzed in this study.

### CRISPR/Cas9-based generation of Suox KOs in C57BL/6N mice

CRISPR KOs were generated through a modified protocol from Chu et al. (60). Cas9 sgRNA was predicted using Benchling software (https://www.benchling.com/) and selected for highest efficiency and lowest off-target rate on chromosome 10. The selected sgRNA (5′-CCTTACTCATTTCGGAGCGCCGG-3′) was tested on a purified PCR amplicon corresponding to a region in the *Suox* Moco domain in an in vitro approach by incubating 30 nM amplicon, 30 nM sgRNA, and 30 nM Cas9 protein (NEB) for 1 h at room temperature (RT) and analyzing cleavage products on a 2% agarose gel. The main process of generating this model was performed by the CECAD research facility, University of Cologne. Fertilized oocytes obtained from breeding of C57BL/6N mice were microinjected with *Suox* guide RNA and *Cas9* mRNA and protein. The oocytes were implanted in pseudo-pregnant C57BL/6N mice. Genotypes of offspring were identified by isolating DNA using QuickExtract buffer (Epicentre; QE09050), subsequently amplifying the targeted area using primers G1 and G2, and employing the T7 endonuclease assay. In short, the PCR amplicon was isolated, and heteroduplex formation was facilitated by incubating the samples at 95°C for 10 min, decreasing the temperature to 85°C at –2°C/min, and finally to 25°C at –0.3°C/min using a PCR cycler. Eighteen microliters of heteroduplexed DNA and 2 U T7 endonuclease I (IDT) was incubated at 37°C for 1 h. Cleavage of heteroduplex DNA was analyzed on a 2% agarose gel. Target genomic areas of founder mice appearing positive in the T7E1 assay were amplified by PCR, inserted into the pJET vector system (K1232; Thermo Fisher Scientific), and used for transformation into *E*. *coli* DH5α; 24 clones were isolated and grown in 5 mL LB medium, and plasmid DNA was isolated and subsequently sequenced using Sanger sequencing (Eurofins Genomics) using a T7 promoter primer. Founder mice harboring the desired mutation were bred for 2 generations before heterozygous matings were started to ensure proper separation of genotypes from chimeric mice.

### Mice-keeping, Suox-KO mouse breeding, and scoring

Mice were kept under a 12 h light cycle and provided with regular chow diet and water *ad libitum*. For the generation of homozygous *Suox*-KO mice, heterozygous mice of at least 2 months of age were kept in 1:1 or 1:2 (male/female) breedings. In case of 1:2 breedings, other females than the mother were removed from the cage before the litters were born. The date of birth (P0) was noted, and new litters were observed twice a day until the litter was terminated. Starting at P4, pups were weighed and phenotypically scored. Animals were sacrificed when weight gain stagnated or animals were found to be suffering. Additionally, righting reflexes of pups were analyzed following a protocol from Didonato and Bogdanik (61). Pups were fixated on their backs for approximately 5 s, and time to right themselves was recorded. Scores according to righting quality were assigned: 3, pup rights itself with correct paw placement; 2, pup rights itself, at least 1 paw is misplaced; 1, pup does not right itself, but struggles to for 60 s; 0, pup does not right itself and aborts struggle within 60 s. Genotyping was performed using tail tips and primers after sacrifice. Genomic DNA was extracted using 50 μL QuickExtract DNA Extraction Solution (Lucigen, Qiagen). DNA sequences surrounding the deletion site were amplified using the following primers: 5′-TAACACATCACAGAGCCGGG-3′ and 5′-CTGGACCCACACACCTATCG-3′. Annealing temperature was 62°C. PCR products were incubated at 37°C for 1 h with the restriction enzyme Bfol (FastDigest Bfol; FD2184; Thermo Fisher Scientific). The products were run in 2% TBE gel for 30 min at 100 V.

### Western blot analysis

Western blotting was performed on crude protein extracts from different lysed tissues or cultured cells in 100 mM Tris/Ac, pH 8.0. Tissue samples were weighed before lysis, and lysis buffer volume was adjusted accordingly. Unless otherwise indicated, 15 μg of protein lysate was separated by SDS-PAGE and immunoblotted using standard protocols. Membranes were probed with primary antibodies against SUOX/SO (Abcam; ab57852), GAPDH (Sigma-Aldrich; G9545), SQORL/SQR (Novusbio; NBP1-84510), TST (Abcam; ab166625), and Vinculin (VCL; Cell Signaling; 4650) as well as either α-mouse, HRP-coupled (Sigma-Aldrich; AP181P) or α-rabbit, HRP-coupled (Santa Cruz Biotechnology; sc-2054) secondary antibody. Signals were detected using chemiluminescent substrates (34580; Thermo Fisher Scientific) and a Bio-Rad ChemiDoc XRS+ system. For densitometric measurements, images were converted to 8-bit using ImageJ (version 1.50i; NIH). Lanes were selected using the square tool, and histograms for individual bands were evaluated after background removal. Identical squares were used for each quantified band. Unless otherwise stated, signals were normalized to VCL or GAPDH as loading control.

### SOX activity measurements

SOX activity was measured using the sulfite:cytochrome *c* activity assay in multiple tissues, as described previously (11). In short, tissue samples were removed from animals after sacrifice, flash-frozen in liquid nitrogen, and stored at –80°C until further use. Tissues were lysed in 100 mM Tris/Ac, pH 8.0, using a loose-fitting homogenizer at 1,200 rpm for 10 cycles and subsequent sonication for 60 s at 15% using 3 s/3 s cycles. Lysates were centrifuged at 21,000*g* for 45 min at 4°C. Protein concentration was determined using the Bradford assay. In general, 20–200 μg crude protein extract was incubated in 200 μL 50 mM Tris/Ac, pH 8.0, 0.2 mM deoxycholic acid, 0.1 mM potassium cyanide, and 0.5 mM sulfite. The reaction was started by the addition of 100 μL of 100 mM equine cytochrome *c*. SOX activity was determined by monitoring the absorption change at 550 nm (ε_550_ = 19,630 M/cm) using a 96 well plate–based approach and a BioTek plate reader (Agilent BioTek) at RT. Determination of Moco was performed by HPLC Form A analysis as previously described (8).

### Immunohistochemistry

*Suox^WT/WT^* and *Suox^KO/KO^* pups (P8) of either sex were anesthetized with 100 mg/kg xylazine (2% Rompun; Bayer) and 20 mg/kg ketamine (Ketaset; Zoetis) and perfused first with PBS and then 4% (w/v) formaldehyde in PBS. Brains were postfixed for 24 h in 4% formaldehyde/PBS. Afterward, brains were washed with 50 mM NH_4_Cl in PBS and cryoprotected first in 15% and then 30% (w/v) sucrose in PBS. Free-floating coronal slices of the right hemisphere (30 μm thickness) were prepared on a Leica CM3050S cryostat at –16°C and placed in PBS at 4°C. All subsequent incubation and wash steps were performed at RT.

After blocking/permeabilization for 1 h with 10% goat serum and 0.4% Triton X-100 in TBS, the following primary antibodies were used: anti-NeuN specific (1:500; Thermo Fisher Scientific; PA5-78639) and anti-MAP2 specific (1:1,000; Millipore; AB5622). The following secondary antibody was used: goat anti-rabbit Alexa Fluor 647 (1:500; A-21245; Thermo Fisher Scientific). Nuclei were stained with 3 μM DAPI in PBS for 5 min at RT. Slices were mounted with Mowiol/Dabco (Carl ROTH).

### Confocal microscopy and image analysis

Images were acquired on a Leica TCS SP8 LIGHTNING upright confocal microscope, equipped with hybrid detectors (Leica HyD) and the following diode lasers: 405, 488, 552, and 638 nm. Image tiles of entire slices (per tile: 1,024 × 1,024 [1,163.34 μm × 1,163.64 μm]) were recorded with an HC PL APO CS2 ×10/0.40 dry objective and high-magnification images (3,432 × 3,432 [144.77 μm × 144.77 μm]) with an HC PL APO CS2 ×63/1.30 GLYC objective. LIGHTNING adaptive deconvolution using the Mowiol setting was used for high-magnification images. Images were segmented and analyzed in an automated fashion using ImageJ/FIJI 1.53c (https://imagej.net/software/fiji/). Rolling ball background subtraction, median filtering, and Otsu auto thresholding methods were used for image segmentation.

### Sulfite, thiosulfate, and cysteine determination in plasma and urine

Plasma was collected by mixing whole blood with EDTA and centrifuged. Both plasma and urine were stored at –80°C until further measurement. Sulfite and thiosulfate were determined using monobromobimane derivatization and subsequent HPLC analysis (29). Fifteen microliters of sample was mixed with 15 μL 160 mM HEPES, pH 8.0, 16 mM EDTA, 15 μL acetonitrile (ACN), and 3 μL 46 mM monobromobimane in ACN. The samples were mixed and incubated in the dark for 30 min at RT. The reactions were stopped with either 65 mM methanesulfonic acid (for urine) or 1.5% methanesulfonic acid (for plasma). The samples were diluted with a 4-fold volume of solvent A (0.5% acetic acid, pH 5.0). Flow rate was set to 1 mL/min. Sample mix (5 or 80 μL) was injected into a LiChrospher 60 RP-Select B (5 μm) 125-4 column. The program is described with percent buffer B (100% MeOH): 0.0 min, 8.0%; 7.5 min, 8.0%; 7.51 min, 12.0%; 17.5 min, 12.0%; 17.51 min, 100.0%; 19.0 min, 100%; 19.01 min, 8.0%; and 25.0 min, 8.0%. The sulfite-bimane peak eluted at 3.4 min, the thiosulfate-bimane peak at 6.8 min, and the cysteine-bimane peak at 4.1 min with fluorescent detection at 380 nm excitation and emission at 480 nm.

### SSC determination in plasma and urine

SSC was determined as described previously (25). Protein in plasma samples was precipitated through addition of 2 volumes of ice-cold methanol. Samples were incubated on ice for 15 min and subsequently centrifuged at 21,000*g* for 10 min at 4°C. Urine samples were centrifuged at 21,000*g* for 10 min at 4°C. Fifteen microliters of supernatant from either preparation was mixed with 15 μL 200 mM Na_2_CO_3_/NaHCO_3_, pH 9.5, and 30 μL 20 mM 9-fluorenylmethyloxycarbonyl chloride. The reaction was incubated at RT for 20 min. The reaction was stopped by the addition of 5 μ 80 mM amantadine hydrochloride (dissolved in 1:1 ACN/0.2 M HCl). After 5 min of incubation, the sample was centrifuged at 21,000*g*, and the supernatant was transferred into a glass HPLC vial. SSC was separated on a 250 × 3 mm, 5 μm Nucleodur HTec C18 column (Macherey-Nagel). Solvent A was 50 mM sodium formate and 0.05% trifluoroacetic acid, pH 4.5, in water; solvent B was 100% methanol. Flow rate was set to 1 mL/min; a gradient from 35% to 60% B was performed from 0 to 7.5 min; SSC eluted at 8.81 min SSC peak area was quantified at 265 nm.

### Amino acid quantification in mouse plasma and urine

Amino acids in mouse plasma and urine were measured by an automated column amino acid analyzer (Biochrom 30, Laborservice Onken GmbH), according to standardized procedures (63).

### MRI of mouse brains

A 3.0 T Philips Achieva clinical MRI scanner (Philips Best) in combination with a dedicated mouse solenoid coil (Philips) was used for imaging. Three-dimensional T2-weighted MR images were acquired postmortem using a turbo-spin echo sequence with repetition time = 439 ms, echo time = 48 ms, field of view = 60 × 35 × 25 mm^3^, voxel size = 0.2 × 0.2 × 0.2 mm^3^, and number of averages = 3. MR images were analyzed with the software VINCI 4.72 (Max Planck Institute for Metabolism Research). Brain volume was determined by manually fitting a volume of interest to each brain.

### In vitro and monobromobimane-based H2S detection

NAP-SSH was synthesized as described by Artaud and Galardon (32). TNB-SSH was generated by adding an equimolar amount of NaHS to DTNB in 50 mM PBS, pH 7.4. HSA-SSH and GAPDH-SSH were generated as described previously (33). For the detection of H_2_S release from persulfidated compounds, a selective H_2_S electrode of a Free Radical Analyzer (World Precision Instruments) was used. For detection of bioavailable H_2_S in plasma, monobromobimane labeling followed by HPLC with fluorescent detection was employed based on a standardized method (27). Twenty-five microliters of the plasma sample was mixed with 66 μL of freshly mixed working buffer (65 μL 200 mM HEPES, pH 8.2, and 100 mM monobromobimane in ACN) in the dark at 20°C for 10 min. The reaction was stopped by the addition of 5 μL 50% TCA, precipitated proteins were removed by centrifugation at 3,000*g* for 5 min at RT, and the supernatants were transferred into amber HPLC autosampler vials. Two microliters was injected into a Phenomenex Luna C18(2) column (250 × 2 mm, 3 μm) and separated by the following optimized gradient elution profile using 0.1% TFA/H_2_O (A) and 0.1% TFA/ACN (B) with a 0.2 mL/min flow rate. The gradient started with 15% B increasing to 35% in 3.54 min and kept there for 7.09 min. In 2.36 min, the composition was increased to 90% B, held for 1.18 min, and returned to the initial 15% in 1.18 min. A Thermo Fisher Scientific UltiMate 3000 HPLC system was used for the separation; fluorescence was detected with an excitation wavelength of 390 nm and an emission wavelength of 475 nm.

### Detection of persulfidated proteins in tissue

#### Proteomic analysis of the persulfidome.

Persulfidation labeling was performed as described previously (18). Briefly, liver samples were lysed in cold HEN buffer (50 mM HEPES, 1 mM EDTA, 0.1 mM Neocuproine (Merck), 1% IGEPAL (Merck), and 2% SDS, pH 7.4) supplemented with 10 mM 4-chloro-7-nitrobenzofurazan (Sigma-Aldrich; 163260) and 1% protease inhibitor using TissueLyser II (Qiagen) and 5 mm stainless steel beads (Qiagen; 69989). Lysates were incubated for 2 h at 37°C and protected from light. Methanol/chloroform precipitation was performed twice (MeOH/CHCl_3_/sample, 4/1/1, v/v/v), and pellets were washed with cold methanol. After resuspension in 2% SDS in PBS (Sigma-Aldrich; P4474), samples were cleaned from endogenously biotinylated proteins using Pierce NeutrAvidin Agarose beads (Thermo Fisher Scientific; 29200) and precipitated. Pellets were then resuspended in PBS and 2% SDS, incubated for 1.5 h at 37°C with 250 μM DCP-Bio1 (Merck; NS1226), and precipitated again. Protein concentration was adjusted to the same level using DC protein assay. After 2 h of incubation on a Ferris wheel at RT and protection from light, supernatants were collected and precipitated. Proteins were redissolved, and their concentration was adjusted to the same level using DC protein assay. Equal amounts of proteins were mixed with Pierce High Capacity Streptavidin Agarose beads and incubated at RT for 4 h in a Ferris wheel protected from light. The samples were transferred into 2 mL Pierce Disposable Columns (Thermo Fisher Scientific; 29920) in which the washing step was carried out with 28 mL of PBS and 8 mL of water to remove nonspecifically bound proteins and detergent. The elution step of enriched proteins was performed using 2.25 M ammonia solution (Sigma-Aldrich; 5.33003) for 16 h. Samples were lyophilized and dissolved in digestion buffer (50 mM ammonium bicarbonate and 1 mM calcium chloride) and digested overnight using trypsin (Promega; V5117) at a trypsin/protein ratio of 1:20. The desalting step was performed on a Supel-Select HLB SPE Tube (Sigma-Aldrich; 54181-U).

Peptides were dissolved in 0.1% TFA, and digestion quality control was performed using an UltiMate 3000 Nano Ultra-High-Pressure Chromatography (UPLC) system with a PepSwift Monolithic Trap (200 μm × 5 mm; Thermo Fisher Scientific). Peptides were analyzed by high-resolution liquid chromatography–tandem mass spectrometry (LC-MS/MS) using an UltiMate 3000 UPLC system coupled with a Thermo Orbitrap Eclipse Tribrid mass spectrometer via an EASY-Spray (Thermo Fisher Scientific). Peptide separation was carried out with an Acclaim PepMap 100 C18 column (Thermo Fisher Scientific) using a 120 min nonlinear gradient from 3% to 35% B (0 min, 3% B; 5 min, 7% B; 120 min, 35% B; A: H_2_O, B: 84% acetonitrile and 0.1% formic acid) at a flow rate of 250 nL/min. The Orbitrap Eclipse was operated in data-dependent acquisition (DDA) mode, and MS1 survey scans were acquired from 300 to 1,500 *m*/*z* at a resolution of 120,000 using the Orbitrap mode. The 10 most intense peaks with a charge state ≥ 2 were fragmented using higher-energy collisional dissociation (HCD) at 32% and analyzed using an ion trap at a normal scan rate. The dynamic exclusion was set at 30 s.

Data were evaluated with PEAKS ONLINE software using 15 ppm for precursor mass tolerance, 0.5 Da for fragment mass tolerance, specific tryptic digest, and a maximum of 3 missed cleavages. NBF (+163.00961594 Da) on C, K, R, DCP (+168.0786442 Da) on C, N-term acetylation (+42.010565 Da), and methionine oxidation (+15.994915 Da) were added as variable modifications, and peptide-spectrum match (PSM) and proteins were filtered at 1% FDR. Data were normalized using the eigenMS (64) script in R studio.

#### Total proteome analysis.

For the total proteome analysis, 100 μg of proteins obtained by the above-mentioned lysis step was digested overnight at 37°C using trypsin with a 1:20 trypsin/sample ratio (Promega; V5117). The next day, peptides were desalted and evaporated under vacuum until dryness, as described above.

Peptides were dissolved in 0.1% TFA, and digestion quality control was performed as described above. Peptides were analyzed by high-resolution LC-MS/MS using an UltiMate 3000 UPLC system coupled with a Thermo Orbitrap Eclipse Tribrid mass spectrometer via an EASY-Spray. Peptide separation was carried out with an Acclaim PepMap 100 C18 column using a 150 min linear gradient from 3% to 35% of B (0 min, 3% B; 140 min, 28% B; 150 min, 35% B; A, H_2_O; B, 84% ACN and 0.1% formic acid) at a flow rate of 250 nL/min. Thermo Orbitrap Eclipse was operated in DDA mode using Thermo Xcalibur Instrument Setup Software. For the MS scan, the detector was operated in Orbitrap with a scan range between 300 and 1,500 with positive polarity, including charge states between 2 and 7. For the MS2, we used quadrupole isolation mode with a HCD activation type and a dynamic exclusion parameter of 30 s for the exclusion duration.

Data were evaluated with PEAKS Online software (Bioinformatics Solutions Inc.) using 15 ppm for precursor mass tolerance, 0.5 Da for fragment mass tolerance, specific tryptic digest, and a maximum of 3 missed cleavages. Carbamidomethylation (+57.021464 Da) on C, N-term acetylation (+42.010565 Da), and methionine oxidation (+15.994915 Da) and NBF (+163.00961594 Da) on C, K, R, were added as variable modifications. PSM and proteins were filtered at 1% FDR. Data were normalized based on the total ion count.

### Proteomic analysis of tissues

Whole tissues were excised from mice after decapitation, washed with PBS, and flash-frozen until processing. For homogenization, tissues were ground in a liquid nitrogen–cooled mortar to fine powder. The tissue powder was suspended in 50 mM triethylammonium bicarbonate and 8 M urea and supplemented with protease inhibitor (cOmplete EDTA-free; Roche) and phosphatase inhibitor (PhosSTOP; Roche). Chromatin was degraded using a Bioruptor sonication rod (Diagenode Diagnostics) for 10 min using 30 s/30 s pulse-break cycles on ice. After centrifugation and protein concentration determination, 50 μg protein was transferred to a new Eppendorf tube and reduced via addition of DTT (5 mM, 37°C, 1 h). Alkylation was performed by addition of chloroacetamide (40 mM, 37°C, 2 h). Proteolytic cleavage was performed by an initial addition of lysyl endopeptidase C (37°C, 4 h) and subsequent addition of trypsin (37°C, overnight). Peptides were purified by mixed chromatography using styrene divinylbenzene-RP. In short, 2 disks of styrene divinylbenzene-RP sulfonate were stacked in pipette tips (StageTips) and equilibrated by consecutive application of (a) methanol, (b) 0.1% formic acid in 80% ACN, and (c) 0.1% formic acid twice. The sample was applied and washed multiple times using (a) 0.1% formic acid and (b) 0.1% formic acid in 80% ACN thrice. The pipette tips were dried and stored at 4°C until further processing. Analysis was conducted by the CECAD Proteomics Core Facility. In short, peptides were separated using C18-RP chromatography (Poroshell EC120; Agilent; 50 cm length, 75 μm diameter, 2.7 μm particle size) on an EASY nLC (Thermo Fisher Scientific). Peptides were loaded with solvent A (0.1% formic acid) and separated at 250 nL/min in a gradient: 3%–4% solvent B (0.1% formic acid in 80% ACN) within 1.0 min, 4%–27% solvent B within 119.0 min, 27%–50% solvent B within 19.0 min, and 50%–95% solvent B within 1.0 min. The mass spectrometer was operated in DDA mode.

### Statistical analysis and data visualization

Individual data points per mouse (5 animals per genotype) and means are displayed in the figures. Data were analyzed using an unpaired 2-tailed Student’s *t* test. Statistical analyses and data visualization were performed using R version 4.1.0 (5/18/2021), rstatix v. 0.7.0, RcolorBrewer v. 1.1-2, dplyr v. 1.0.7, purrr v. 0.3.4, readr v. 2.0.0, tidyr v. 1.1.3, tibble v. 3.1.3, ggplot2 v. 3.3.5, tidyverse v. 1.3.1, rio v. 0.5.27, and pacman v. 0.5.1 in the RStudio environment (https://www.rstudio.com/), platform x86 64-w64-mingw32/x64 (64-bit). Figures were prepared with OMERO (https://www.openmicroscopy.org/).

### Study approval

All animals were kept and bred in accordance with European, national, and institutional guidelines, and protocols were approved by local government authorities (Landesamt für Natur, Umwelt und Verbraucherschutz Nordrhein-Westfalen, Germany; reference 84-02.04.2014.A372).

### Data and materials availability

All proteomic data are available through PRIDE under code number PXD045896 (project DOI: 10.6019/PXD045896). All data points shown in graphs are available in the Supporting Data Values file.

## Author contributions

CYF, JBK, FN, and GS designed research studies. CYF, JBK, FL, DD, TD, SO, FN, M Mai, EK, TG, JASA, and SYY conducted experiments. CYF, JBK, FL, DD, TD, SO, M Morita, TG, JASA, and SYY acquired data. CYF, JBK, FL, DD, TD, SO, TG, JASA, ATM, EK, TG, JASA, SYY, HE, M Křížková, VK, M Krueger, JBH, UB, PN, MF, and GS analyzed data. M Morita, UB, TA, and MF provided reagents. CYF, JBK, FN, UB, TA, PN, MF, and GS wrote the manuscript.

## Supplementary Material

Supplemental data

Unedited blot and gel images

Supplemental table 1

Supporting data values

## Version 1 11/03/2025

Electronic publication
