# Supplementary material for: Sulfite oxidase deficiency causes persulfidation loss and hydrogen sulfide release
Source: J Clin Invest. 2025 Nov 3;135(21):e181299. doi: 10.1172/JCI181299 (PMC12578401; doi:10.1172/JCI181299)
Supplement: Supplemental data [file jci-135-181299-s327.pdf]

Supplemental Information

Sulfite oxidase deficiency causes persulfidation loss and hydrogen sulfide release

Chun-Yu Fu[1,#], Joshua B. Kohl[1,#], Filip Liebsch[1,], Davide D'Andrea[2], Tamás Ditrói[3], Seiryo Ogata[4], Franziska Neuser[1], Max Mai[1], Anna T. Mellis[1], Emilia Kouroussis[2], Masanobu Morita[4], Titus Gehling[1], José Angel Santamaria-Araujo[1], Sin Yuin Yeo[5], Heike Endepols[6,7,8], Michaela Křížková[9], Viktor Kozich[9], Marcus Krueger[10], Julia B. Hennermann[11], Uladzimir Barayeu[4,12], Takaaki Akaike[4], Peter Nagy[3,13,14], Milos Filipovic[2,15], Guenter Schwarz[1,16]*

[1] Institute of Biochemistry, Department of Chemistry and Biochemistry, University of Cologne, 50674 Cologne, Germany

[2] Leibniz Institute for Analytical Sciences, ISAS, e.V.

[3] Department of Molecular Immunology and Toxicology and the National Tumor Biology Laboratory, National Institute of Oncology, Budapest, 1122, Hungary

[4] Department of Environmental Medicine and Molecular Toxicology, Tohoku University Graduate School of Medicine, Sendai, Japan

[5] University of Cologne, Faculty of Medicine and University Hospital Cologne, Institute of Diagnostic and Interventional Radiology, Cologne, Germany

[6] University of Cologne, Faculty of Medicine and University Hospital Cologne, Institute of Radiochemistry and Experimental Molecular Imaging, Cologne, Germany

[7] University of Cologne, Faculty of Medicine and University Hospital Cologne, Department of Nuclear Medicine, Cologne, Germany

[8] Forschungszentrum Jülich GmbH, Institute of Neuroscience and Medicine, Nuclear Chemistry (INM-5), Wilhelm-Johnen-Straße, 52428 Jülich, Germany

[9] Department of Pediatrics and Inherited Metabolic Disorders, Charles University-First Faculty of Medicine and General University Hospital in Prague, Prague, Czech Republic

[10] Institute of Genetics, Department of Biology and CECAD, University of Cologne, 50674 Cologne, Germany

[11] Center for Pediatric and Adolescent Medicine, University Medical Center Mainz, Mainz, Germany

[12] Max-Planck-Institute for Polymer Research, Mainz, Germany

[13] Department of Anatomy and Histology, HUN-REN–UVMB Laboratory of Redox Biology Research Group, University of Veterinary Medicine, Budapest, 1078, Hungary

[14] Chemistry Institute, University of Debrecen, Debrecen, Hajdú-Bihar County, 4012, Hungary

[15] School of Molecular Biosciences, University of Glasgow, Glasgow, UK

[16] Center for Molecular Medicine Cologne, University of Cologne, 50674 Cologne, Germany

[#]contributed equally

* corresponding author:

Prof. Dr. Guenter Schwarz, Institute of Biochemistry, Zuelpicher Str. 47, 50674 Koeln, e-mail: gschwarz@uni-koeln.de, Phone: +49 221 470 6440

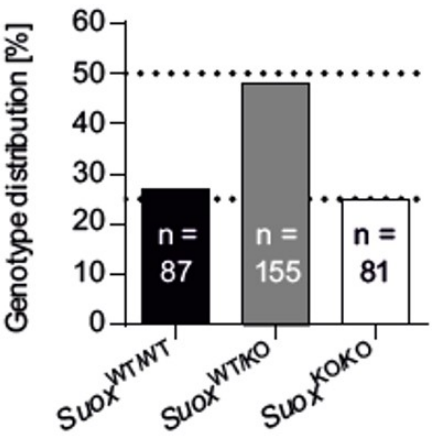

Supplemental Figure 1. Genotype distribution in offspring from *Suox*$^{WT/KO}$ x *Suox*$^{WT/KO}$ breedings.

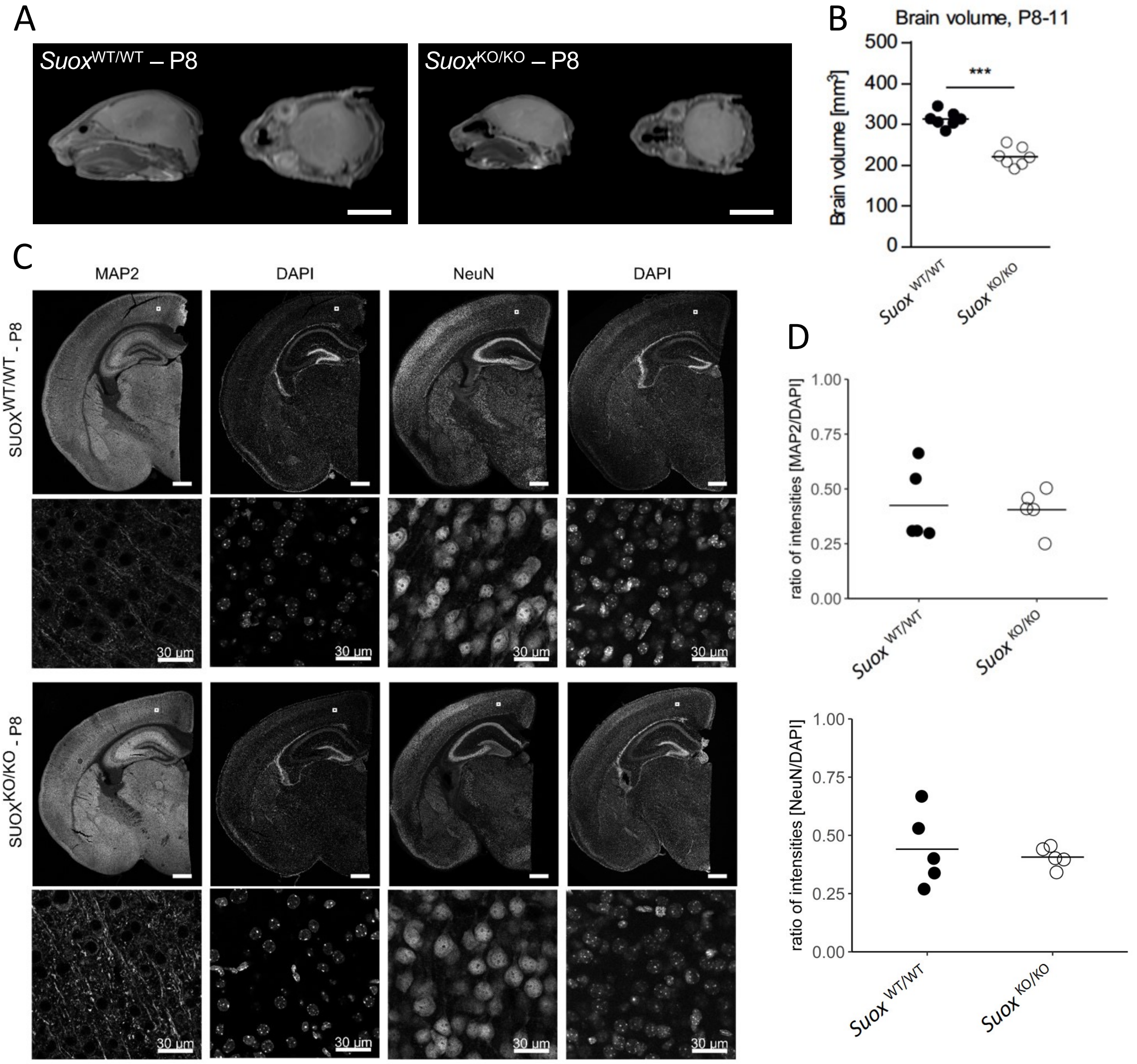

Supplemental Figure 2. Decreased brain volume in *Suox*$^{KO/KO}$ mice, yet no apoptotic neurons were observed.

(A) Brain magnetic resonance imaging (MRI) scans of P8 *Suox*$^{WT/WT}$ and *Suox*$^{KO/KO}$ mice. Scale bar: 5 mm. **(B)** Decreased brain volume of SOX-deficient mice (P8–P11, n = 7 per group, groups are exactly age-matched), p<0.001 (Student's t test). **(C)** IHC staining for microtubule-associated protein 2 (MAP-2) and neuronal nuclear antigen (NeuN). **(D)** Neuron density showed no difference between *Suox*$^{KO/KO}$ and WT mice.

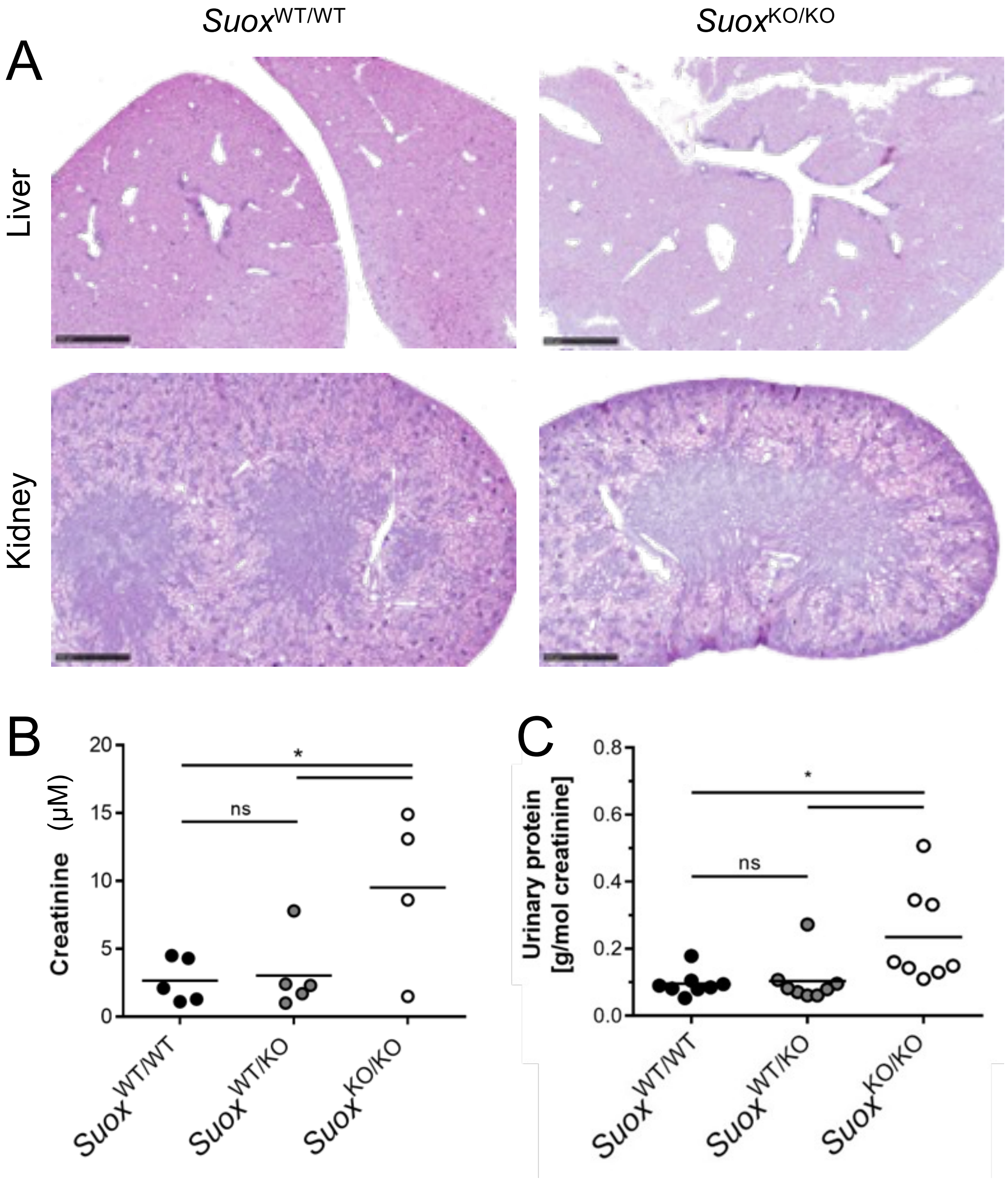

Supplemental Figure 3. Analysis of liver and kidney derived from 5 days old mice WT and *Suox*$^{KO/KO}$

(A) H/A stainings **(B)** Determination of plasma creatinine **(C)** and urinary protein.

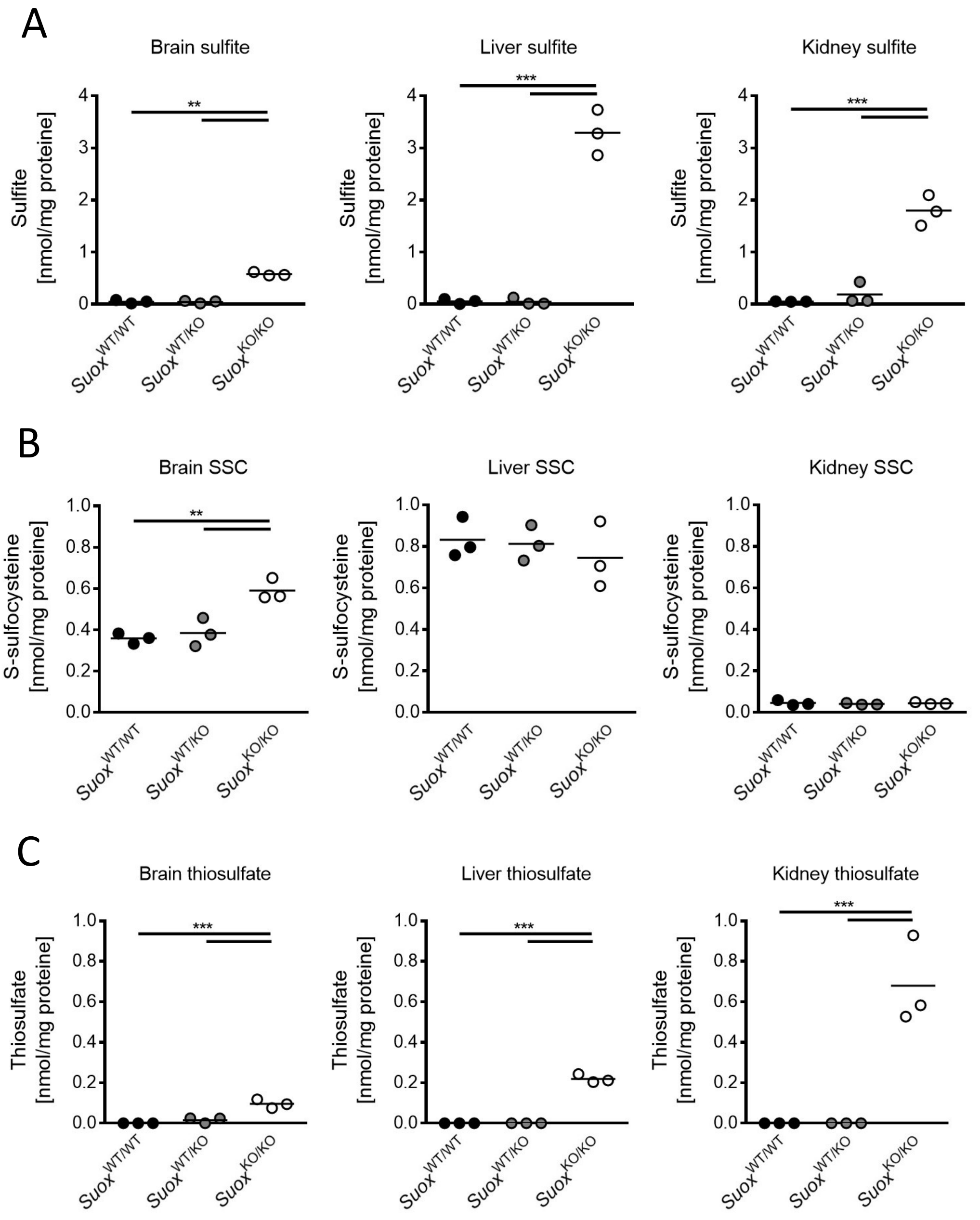

Supplemental Figure 4. Major SOXD biomarkers in tissues in *Suox*$^{KO/KO}$ mice.

(A) Determination of sulfite, **(B)** S-sulfocysteine, and **(C)** thiosulfate in brain, liver and kidney of *Suox*$^{WT/WT}$, *Suox*$^{WT/KO}$, and *Suox*$^{KO/KO}$ mice. One-way ANOVA with Tukey's post-hoc test for pairwise comparisons was performed as indicated. p value: *** < 0.001; ** < 0.01; * < 0.05; ns > 0.05.

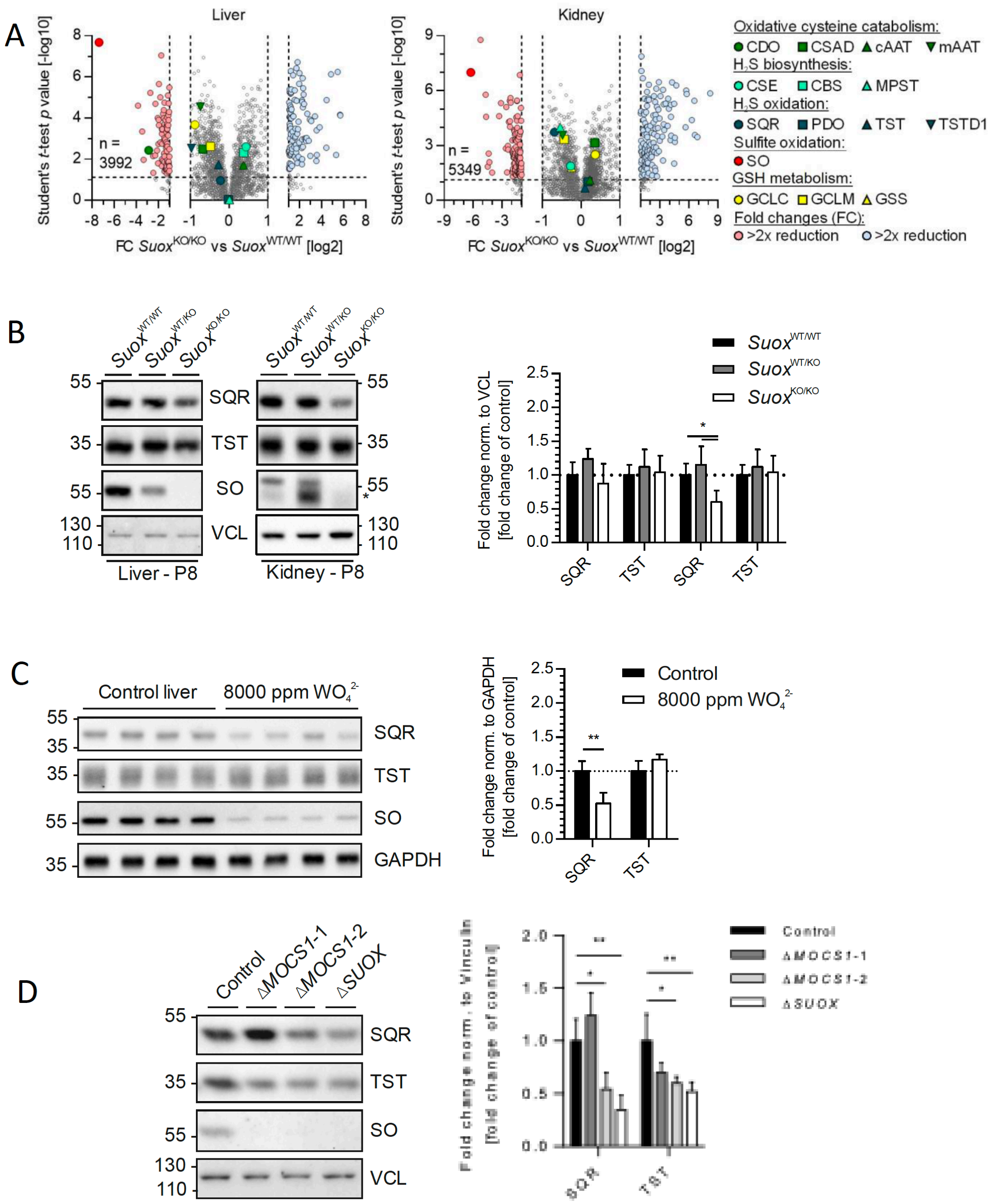

Supplemental Figure 5. Proteomic profiling of of *Suox*$^{KO/KO}$ mice liver and kidney extracts.

(A) Volcano plot depicting significantly regulated proteins of *Suox*$^{KO/KO}$ livers compared to *Suox*$^{WT/WT}$ samples (n = 5). Significantly up-regulated proteins are displayed in blue, significantly down-regulated proteins are displayed in red (absolute log2 fold change ≥ 1, -log10 *p* value ≥ 1.3). Exemplary proteins are labelled with their respective gene names. **(B-D)** Western blot analysis of liver and kidney extracts **(B)**, tungstate-treated HEK293 cells **(C)**, and patient fibroblasts (1-3) (**D**) and the respective densiometric analysis of triplicates (right sight of blots).

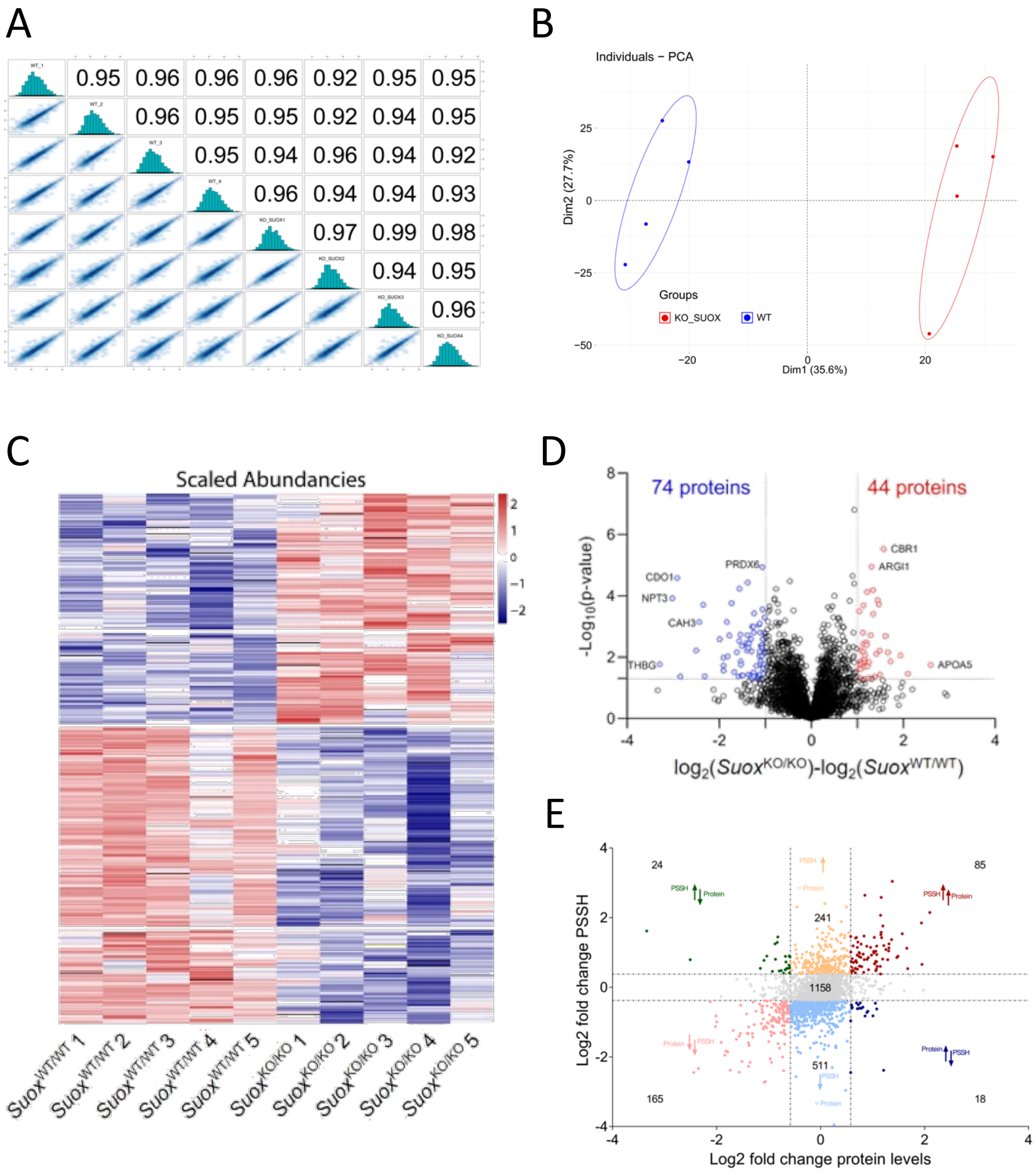

Supplemental Figure 6. Changes in total proteome of *Suox*$^{KO/KO}$ mouse liver extracts. **(A)** Peptide correlation between all measured samples for persulfidome analysis. **(B)** Principal Component Analysis (PCA) shows notable difference in persulfidome of *Suox*$^{KO/KO}$ vs *Suox*$^{WT/WT}$ mice. **(C)** Heatmap showing the changes of protein expression (total proteome) in *Suox*$^{KO/KO}$ mouse livers compared to *Suox*$^{WT/WT}$ mouse (Welch's test, $p<0.05$). **(D)** Volcano plot depicting statistical significance plotted against the $\log_2$-fold change of protein expression (total proteome) in *Suox*$^{KO/KO}$ mouse liver relative to *Suox*$^{WT/WT}$ mouse. Significance was established using Welch's t-test (two-sided), with a p-value threshold of < 0.05. Fold change cut-offs were established at 50%. **(E)** Scatter plot analysis of persulfidome and protein expression changes correlation. Scatter plot of the log2 folds changes within the persulfidome (y-axis) and the protein expression (x-axis) for the comparison between *Suox*$^{KO/KO}$ mice and *Suox*$^{WT/WT}$ mice.

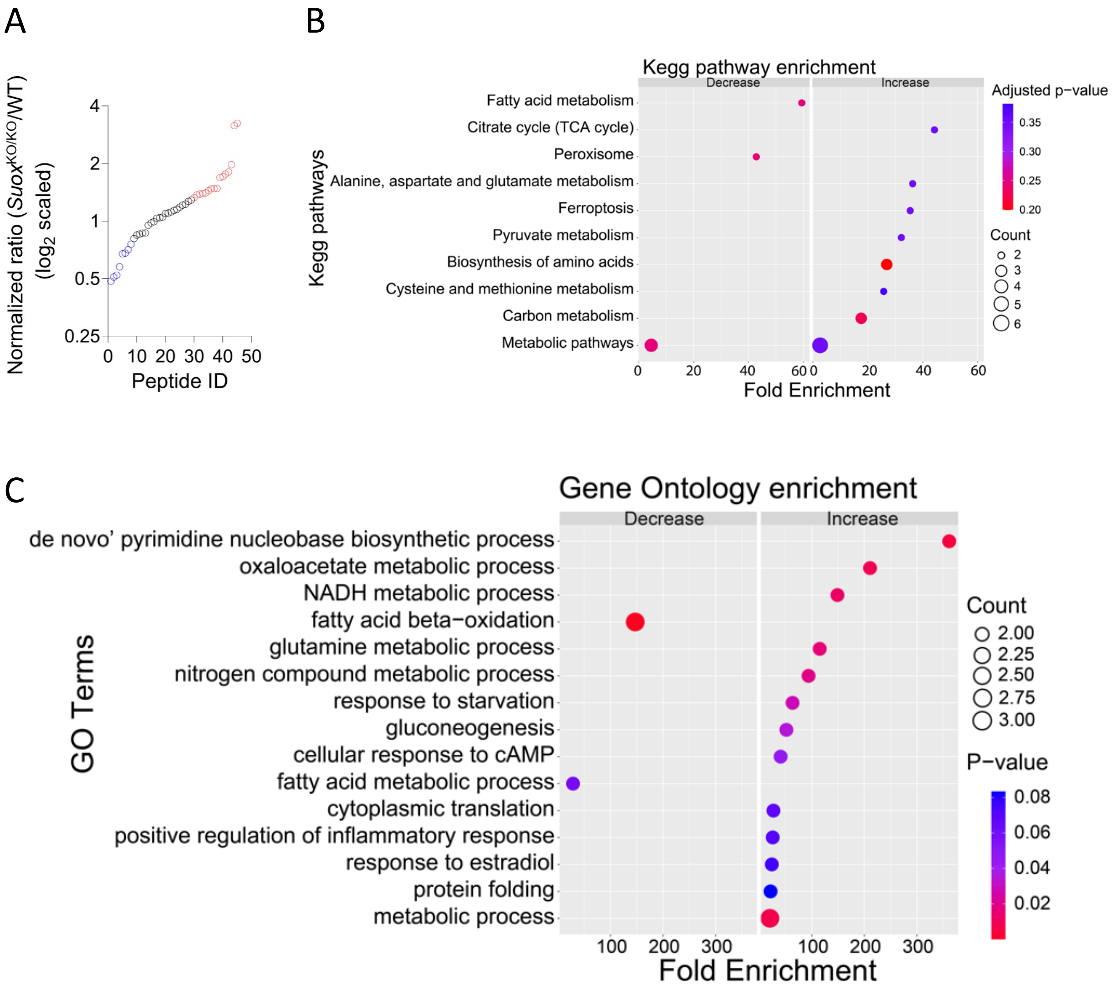

Supplemental Figure 7. Proteome analysis of peptides with S-sulfonylated cysteines within the total proteome of *Suox*$^{KO/KO}$ mouse liver extracts. Based on our *in vitro* studies with persulfidated cysteine, a decrease in protein persulfidation should be mirrored by an increase in S-sulfonylation. However, no method for selective enrichment of this modification exists today. Nonetheless, considering that the first step of the dimedone-switch method is blocking all thiols, persulfides, sulfenic acids and amines, preventing any artificial persulfide oxidation, we looked for the S-sulfonylated peptides in those samples. We could detect 45 peptides passing the inclusion criteria (being present in at least 4 out of 5 samples) corresponding to 40 proteins, of which 40 % showed significant increase in *Suox*$^{KO/KO}$ mice (peptides normalized to actual protein levels, 1.3-fold change) (Suppl. Fig. 6). Despite the low number of identified targets, both Kegg pathway and GO term enrichment analyses pointed towards metabolic processes, such as lipid and amino acid metabolism, as well as ferroptosis, being very similar to the results of the corresponding enrichments analyses in our persulfidation data set. **(A)** $PSSO_3H$ fold change levels normalized to the corresponding protein expression levels. **(B-C)** Kegg pathway (**B**) and GO Term (biological process) (**C**) enrichment analysis of proteins found to significantly decrease or increase their $PSSO_3H$ levels in *Suox*$^{KO/KO}$ mouse liver extracts.

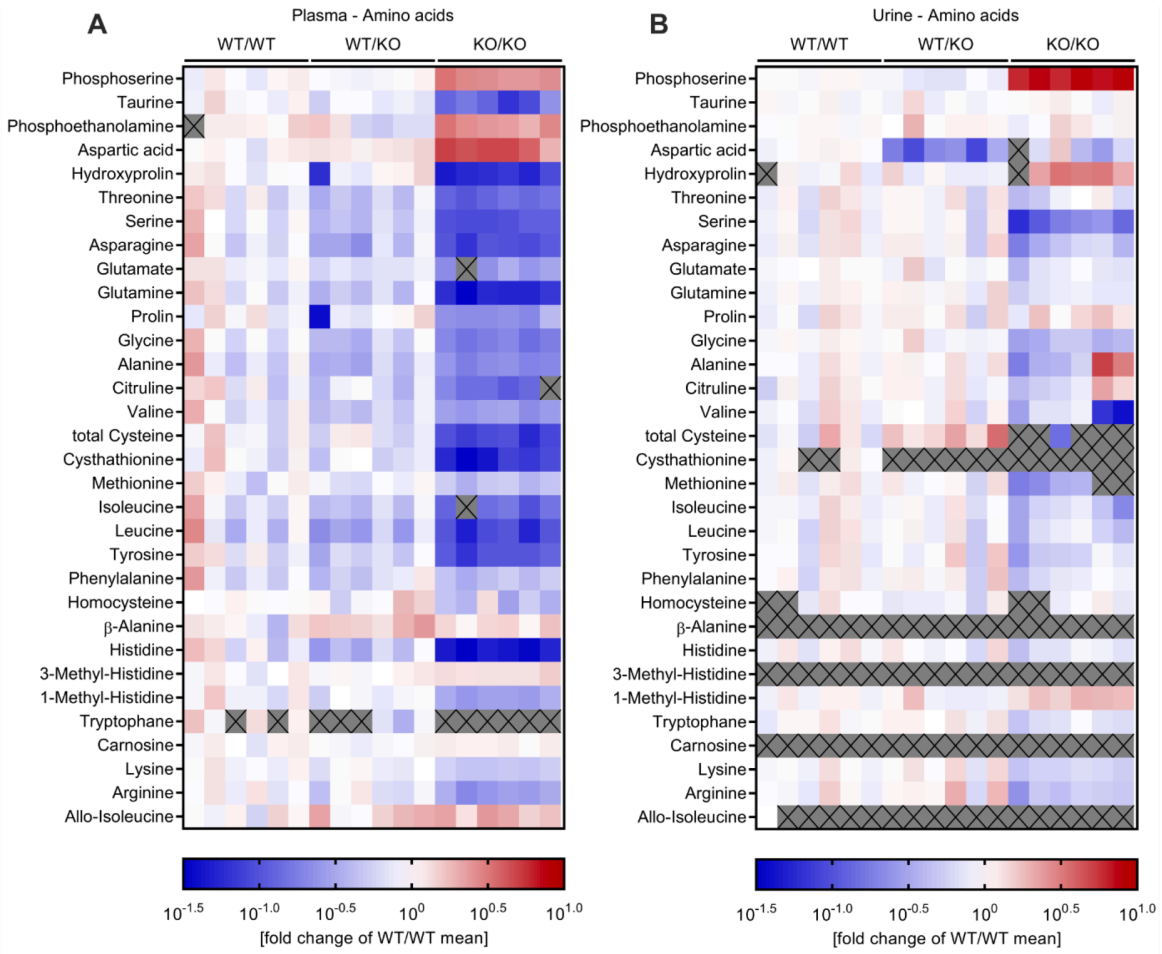

Supplemental Figure 8. Amino acid profile of plasma and urine of SOX-deficient mice. Amino acid profiles of (A) plasma and (B) urine of WT/WT, WT/KO and KO/KO at P8 (n = 6 per genotype), as determined in a ninhydrin-based detection method. Results are displayed as fold changes of the mean of WT/WT values; grey and crossed-out boxes indicate values below the detection limit.

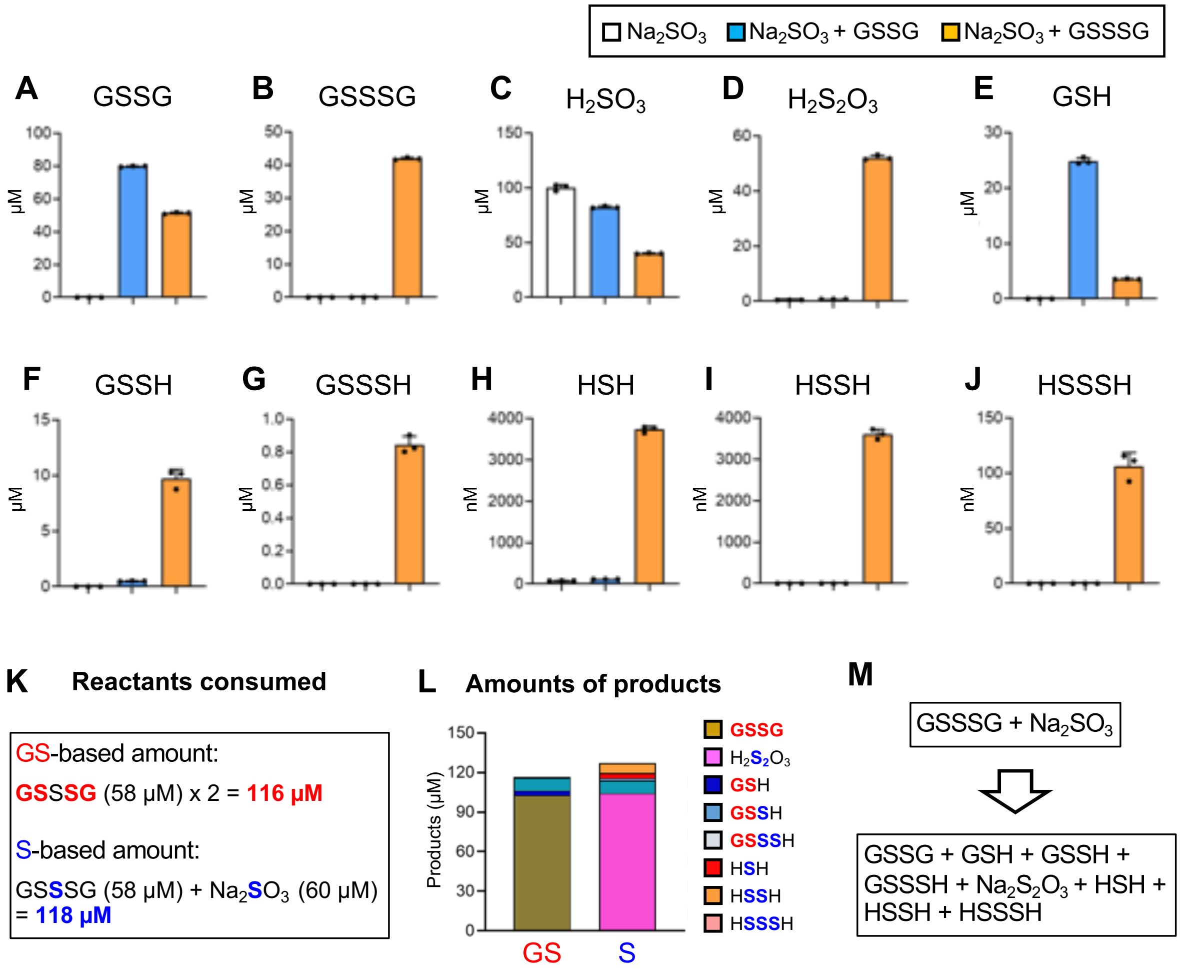

Supplemental Figure 9. In vitro reaction of sulfite (Na_2SO_3) with oxidized glutathione (GSSG) and glutathione trisulfide (GSSSG).

(A-J) Quantification of sulfur metabolites consumed or produced in the reaction mixture of 100 μM Na_2SO_3 with 100 μM GSSG and 100 μM GSSSG. Each metabolite was quantified via LC-MS/MS analysis with b-(4-hydroxyphenyl) ethyl iodoacetamide (HPE-IAM) labeling following a 1 h incubation of the reaction mixture. **(K, L)** Quantitative comparison for the amounts of glutathione (GS)- and sulfur (S)-related compounds consumed **(K)** or produced **(L)**, which were found in the reaction mixture of 100 μM Na_2SO_3 with 100 μM GSSSG. **(M)** The reaction scheme of Na_2SO_3 and GSSSG, which is identified herein.

Supplemental Reference

1. Schwahn BC, Hart C, Smith LA, Hart A, Fairbanks L, Arenas-Hernandez M, et al. cPMP rescue of a neonate with severe molybdenum cofactor deficiency after serendipitous early diagnosis, and characterisation of a novel MOCS1 variant. *Mol Genet Metab.* 2024;143(4):108598.
2. Mayr SJ, Sass JO, Vry J, Kirschner J, Mader I, Hovener JB, et al. A mild case of molybdenum cofactor deficiency defines an alternative route of MOCS1 protein maturation. *J Inherit Metab Dis.* 2018;41(2):187-96.
3. Kaczmarek AT, Bender D, Gehling T, Kohl JB, Daimaguler HS, Santamaria-Araujo JA, et al. A defect in molybdenum cofactor binding causes an attenuated form of sulfite oxidase deficiency. *J Inherit Metab Dis.* 2022;45(2):169-82.
