# Supplementary material for: Sulfite oxidase deficiency causes persulfidation loss and hydrogen sulfide release
Source: J Clin Invest. 2025 Nov 3;135(21):e181299. doi: 10.1172/JCI181299 (PMC12578401; doi:10.1172/JCI181299)

Figure 1C –SO

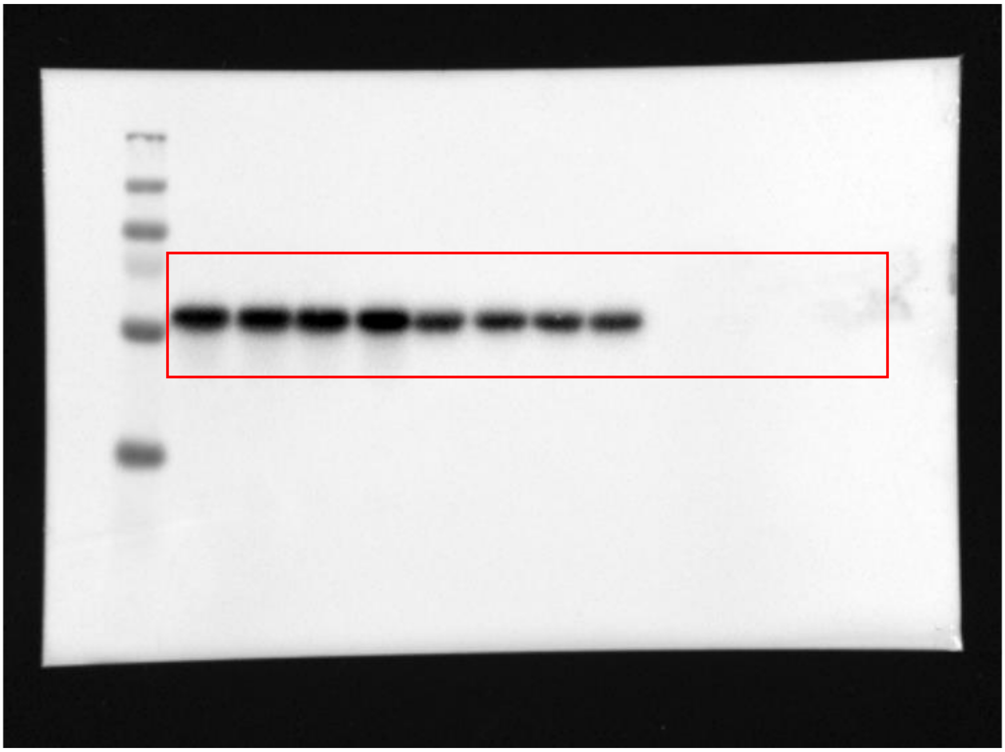

Figure 1C –SO pc

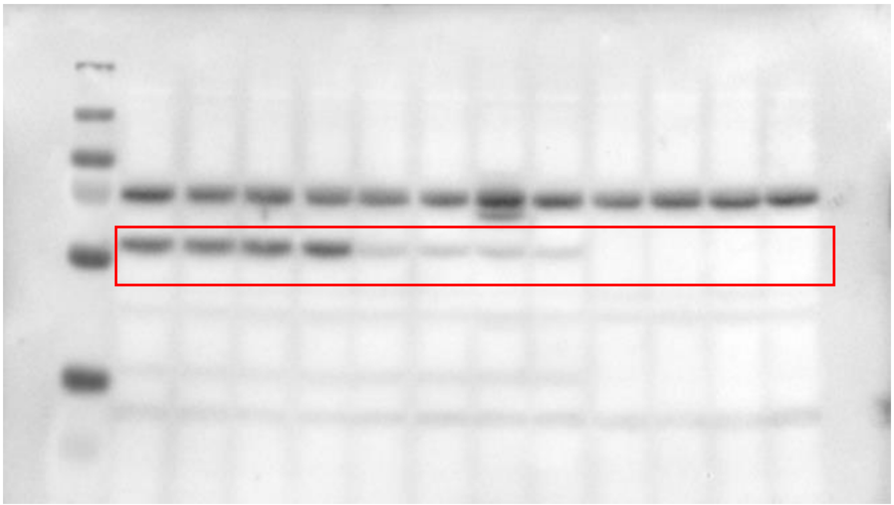

Figure 1C –GAPDH

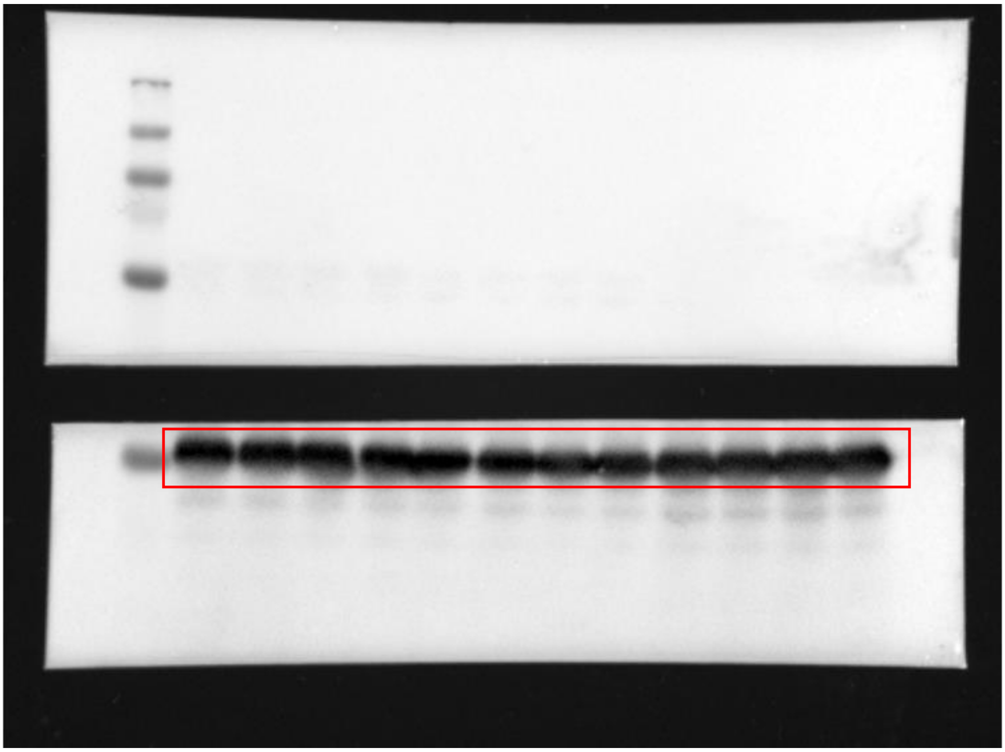

Figure 4B

NBF-Cl

Cy5

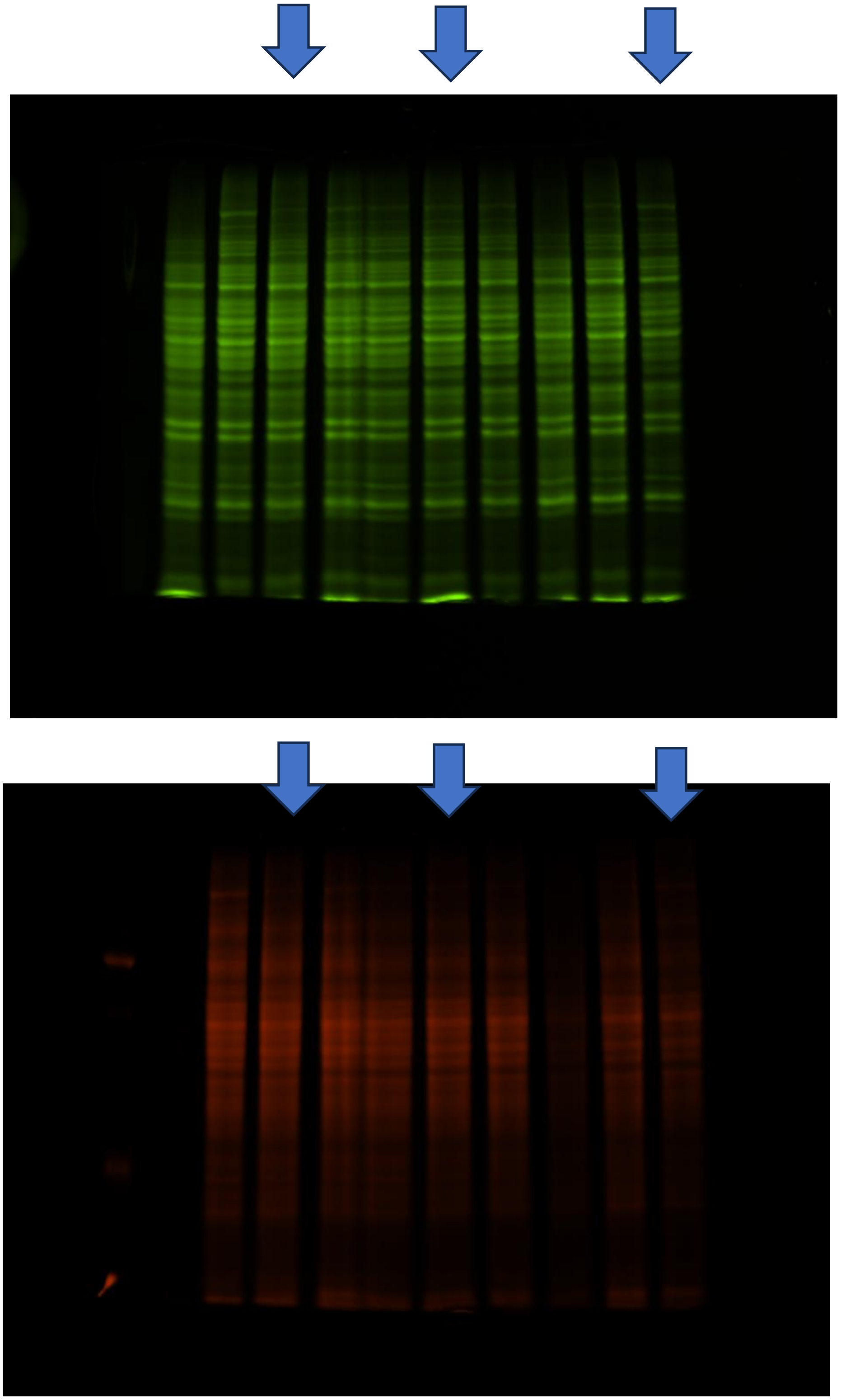

Suppl. Figure 5B – Kidney SQR

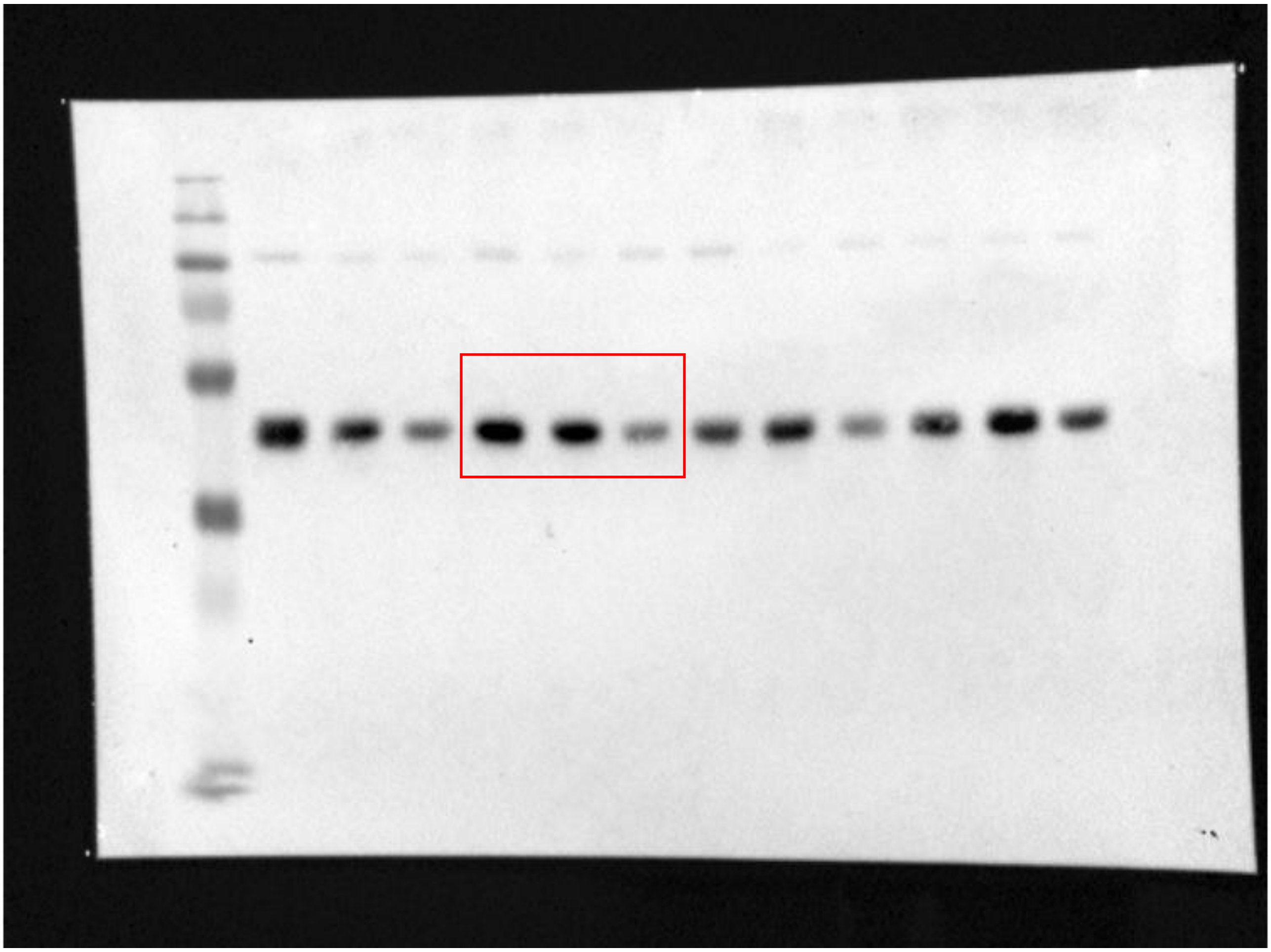

Suppl. Figure 5B – Kidney TST

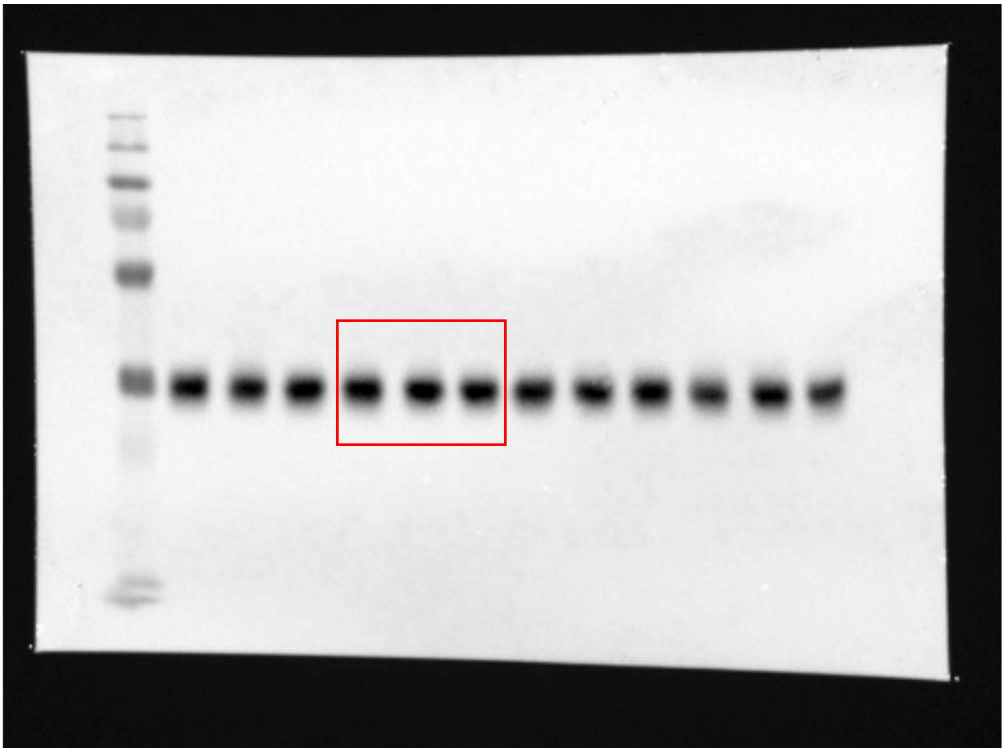

Suppl. Figure 5B – Kidney SO

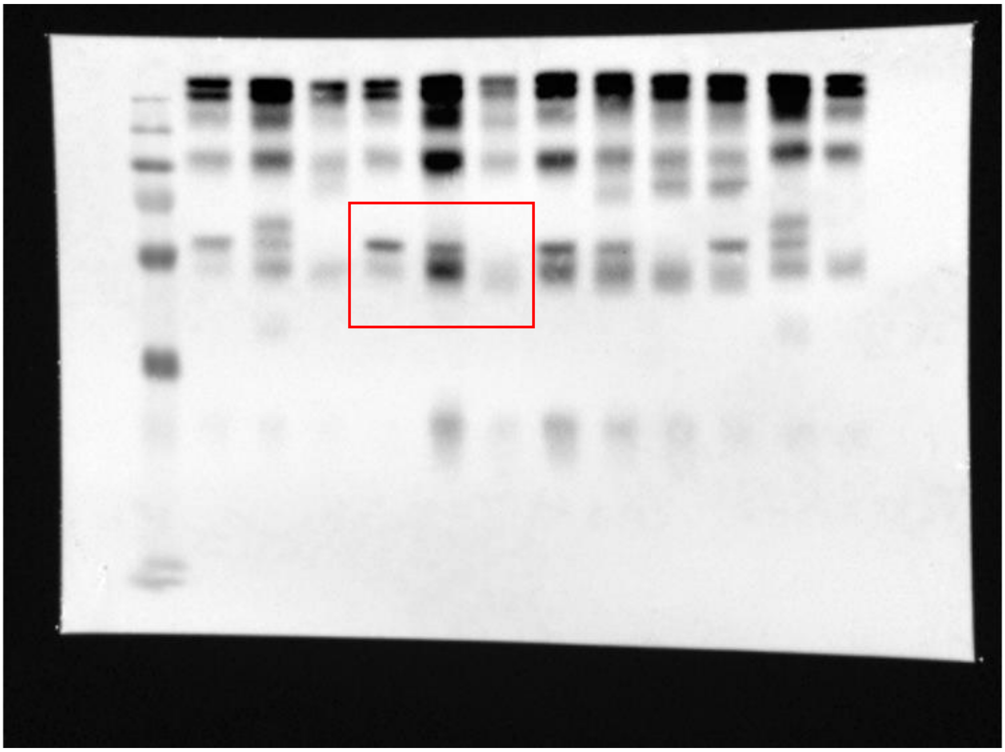

Suppl. Figure 5B - kidney VCL

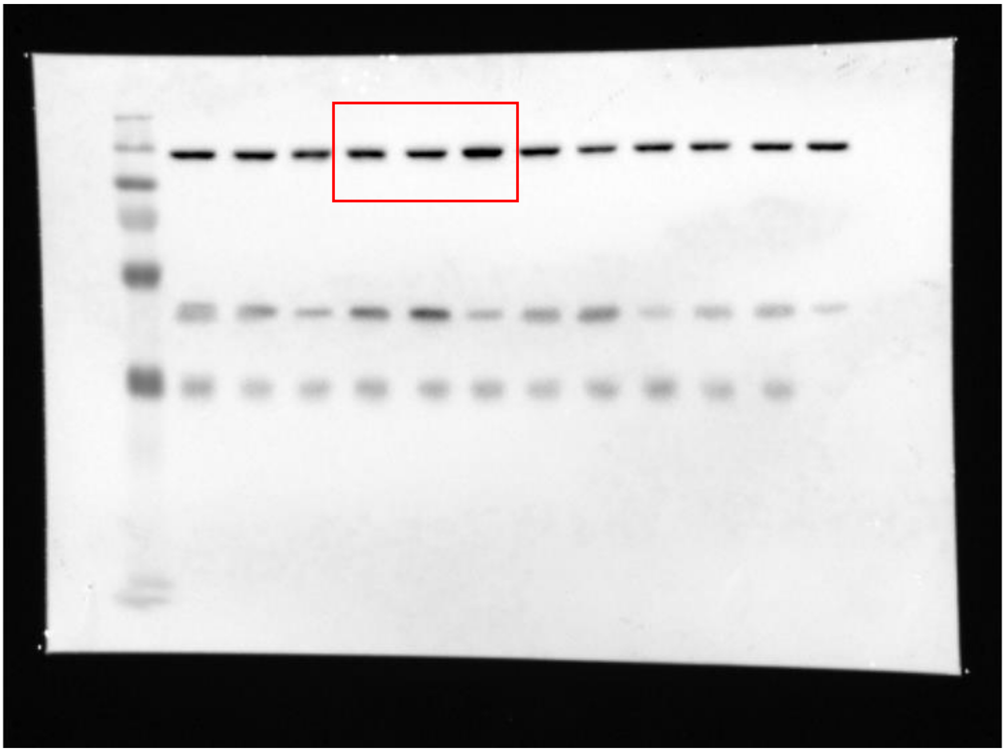

Suppl. Figure 5B – liver SQR

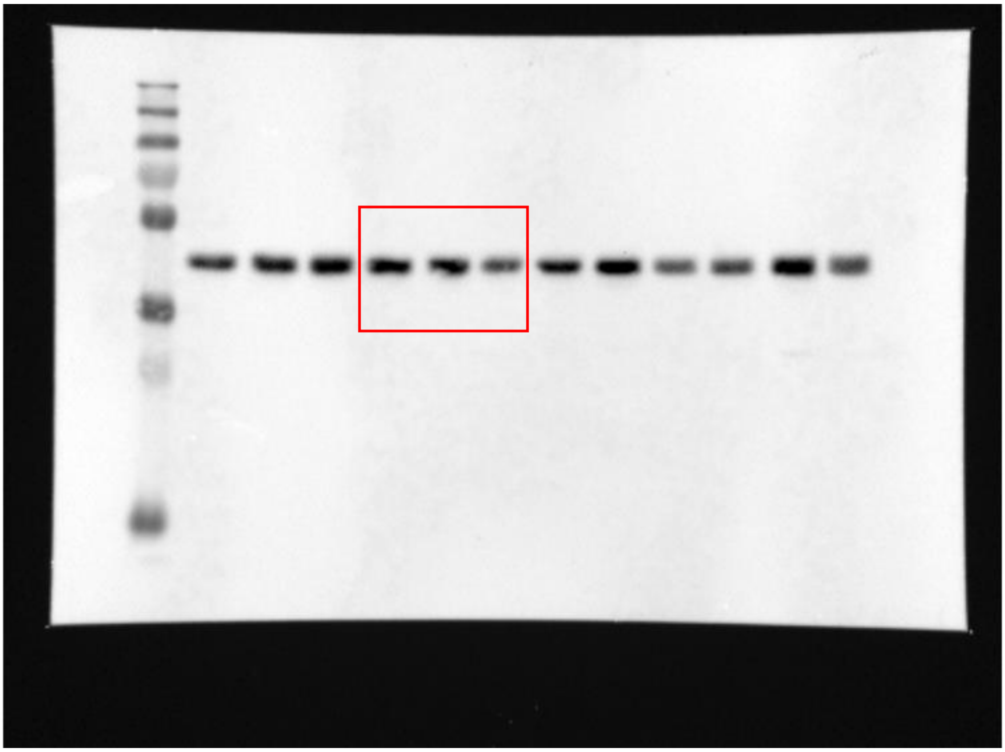

Suppl. Figure 5B – liver TST

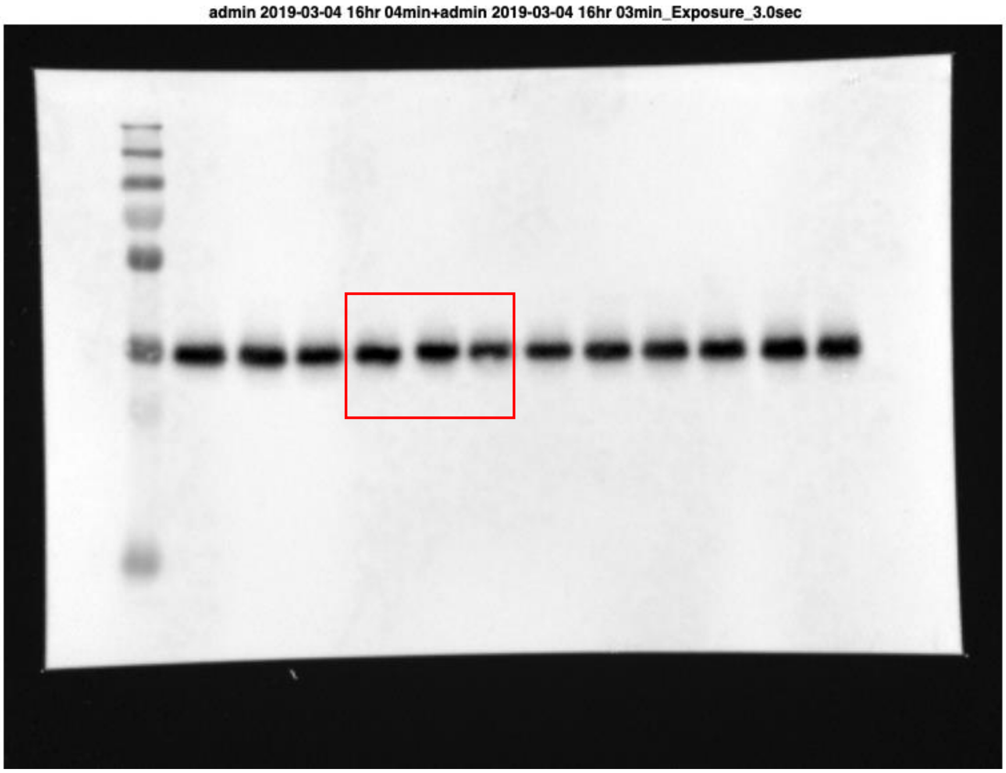

Suppl. Figure 5B – liver SO

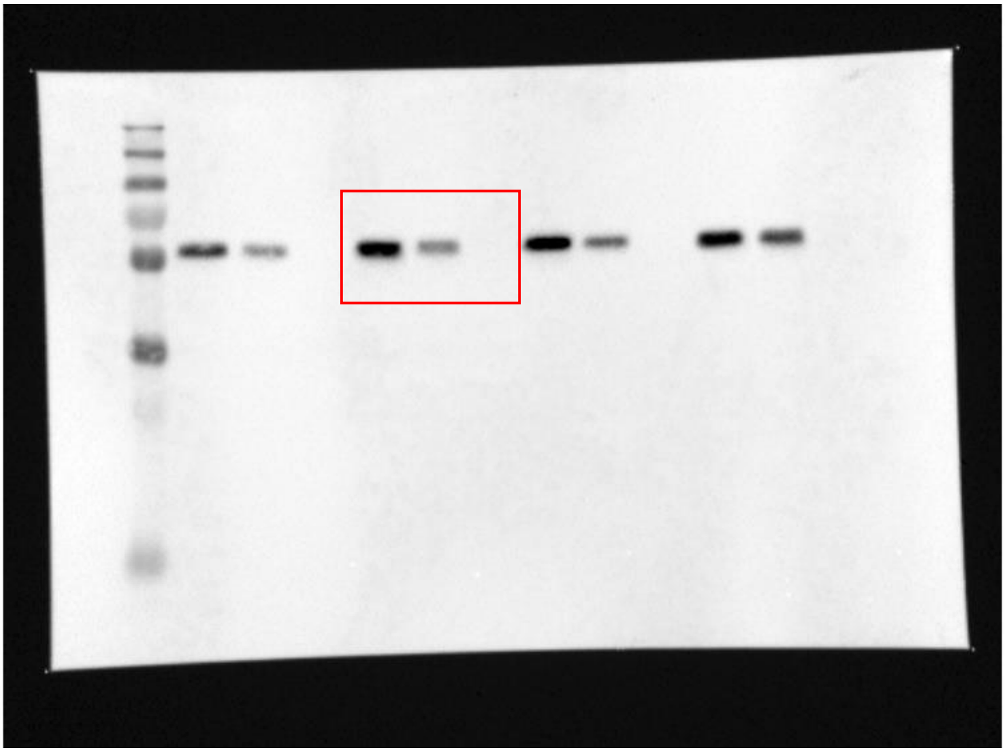

Suppl. Figure 5B - VCL

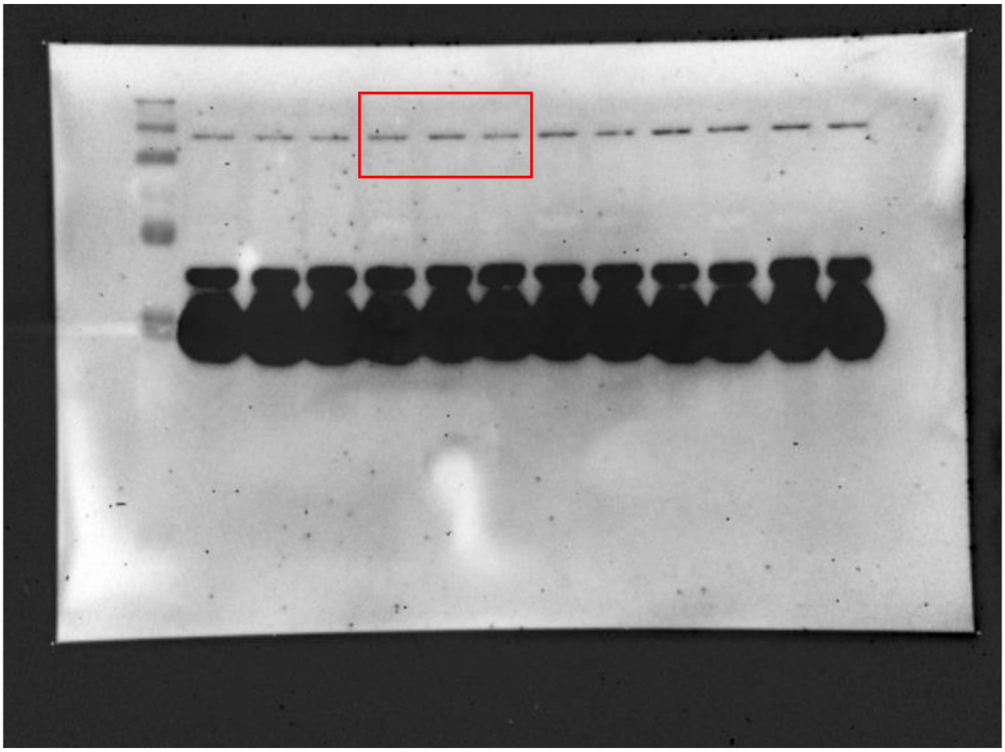

Suppl. Figure 5C SQR

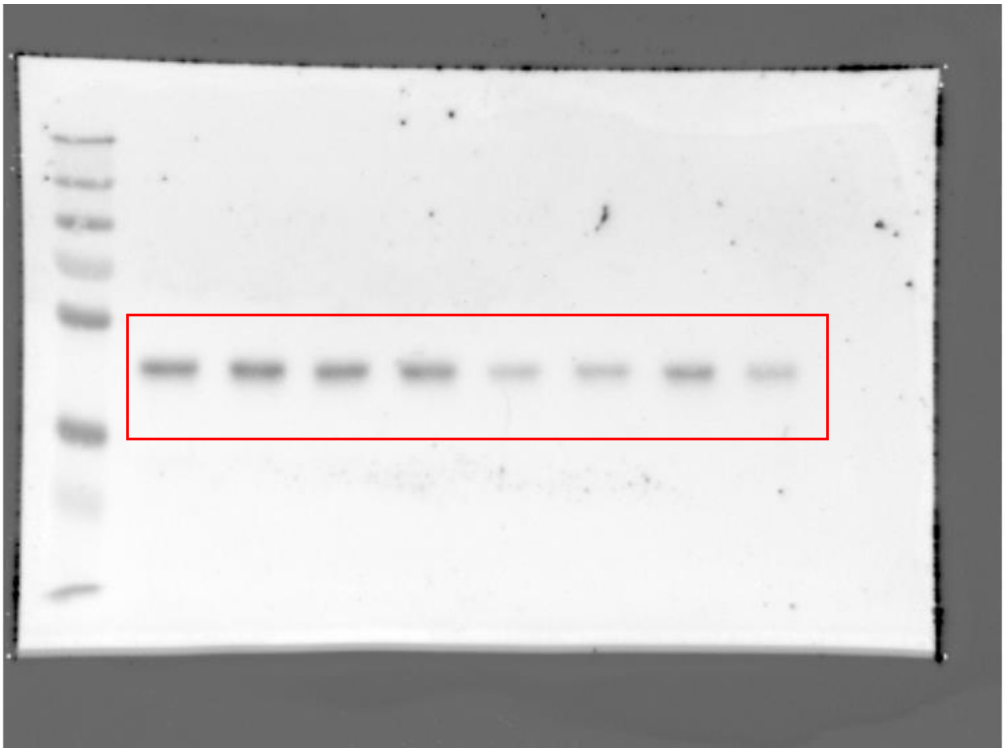

Suppl. Figure 5C TST

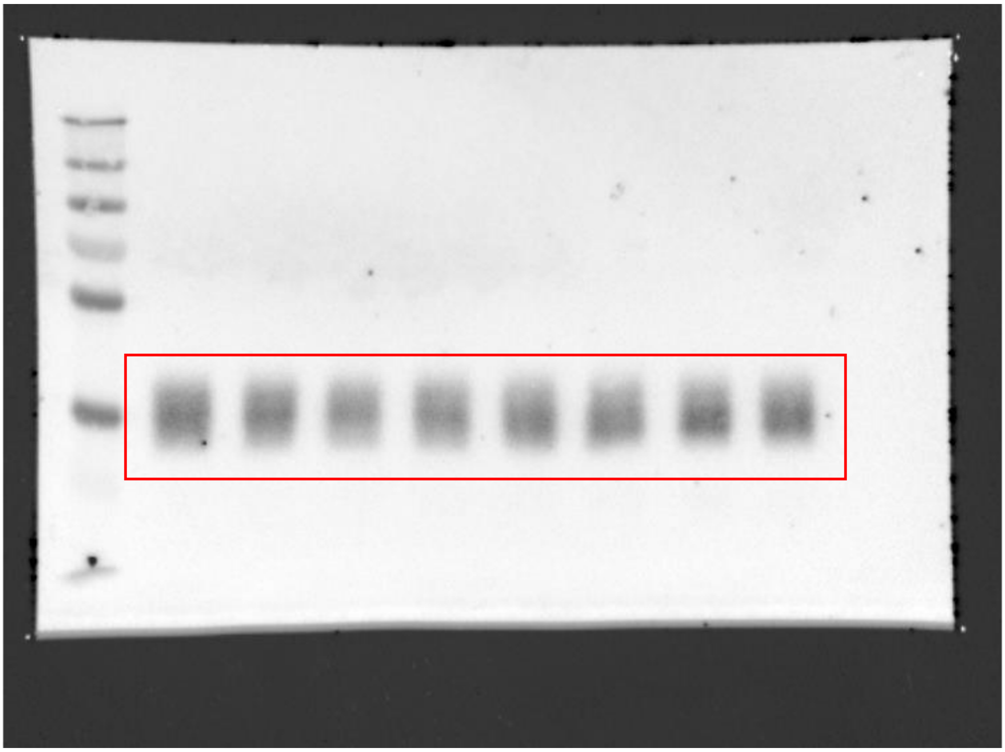

Suppl. Figure 5C SO

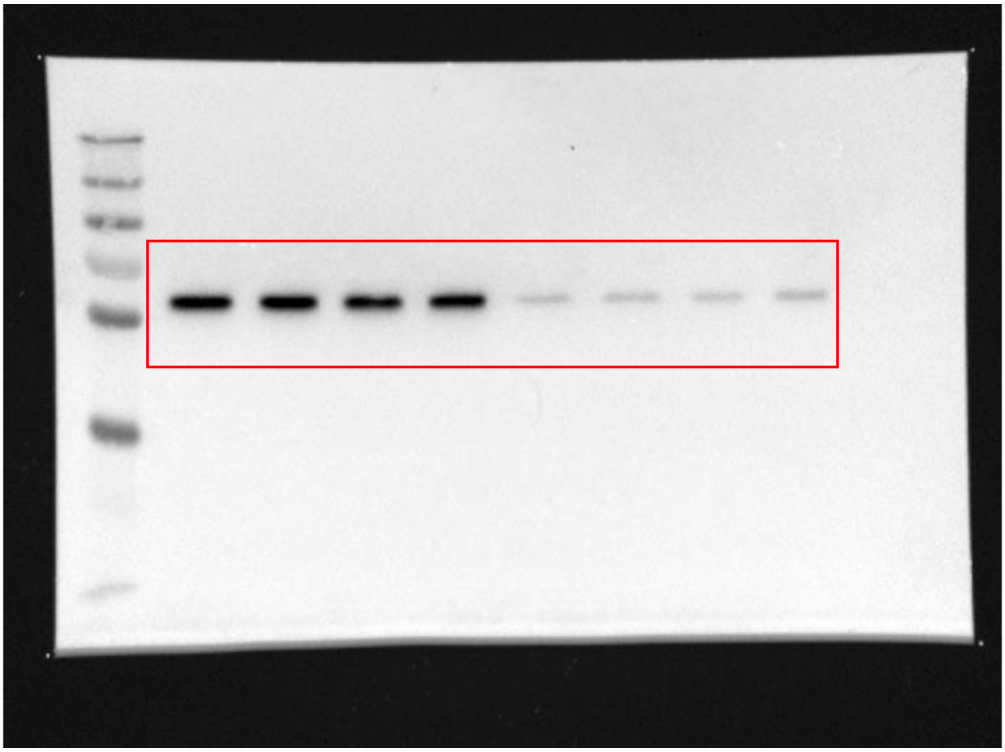

Suppl. Figure 5C GAPDH

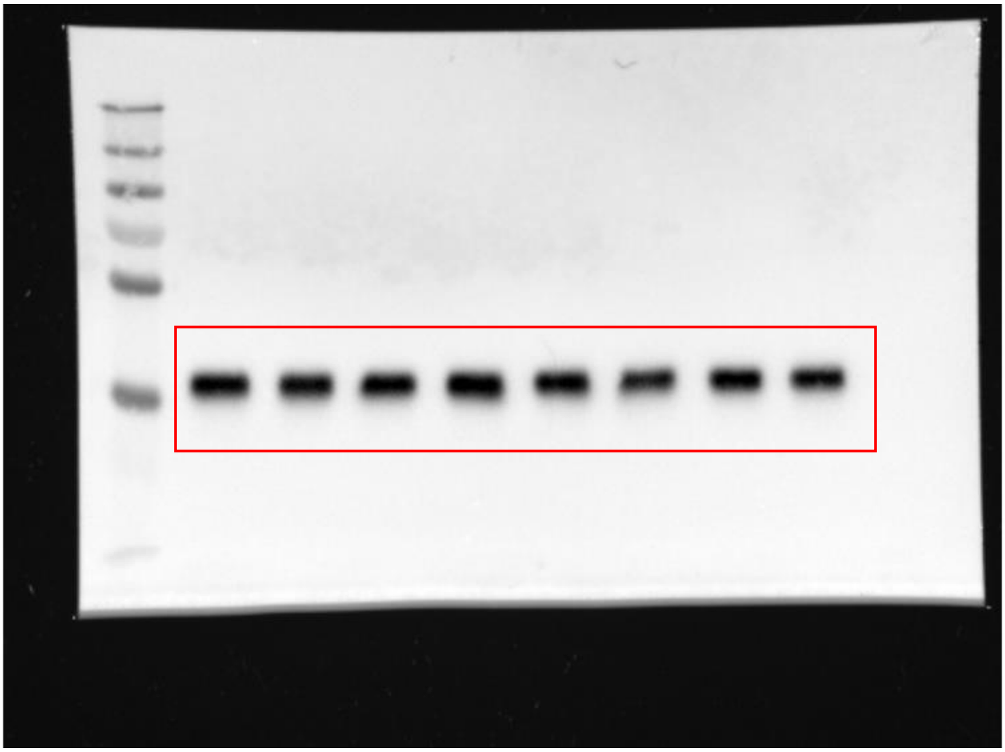

Suppl. Figure 5D SQR

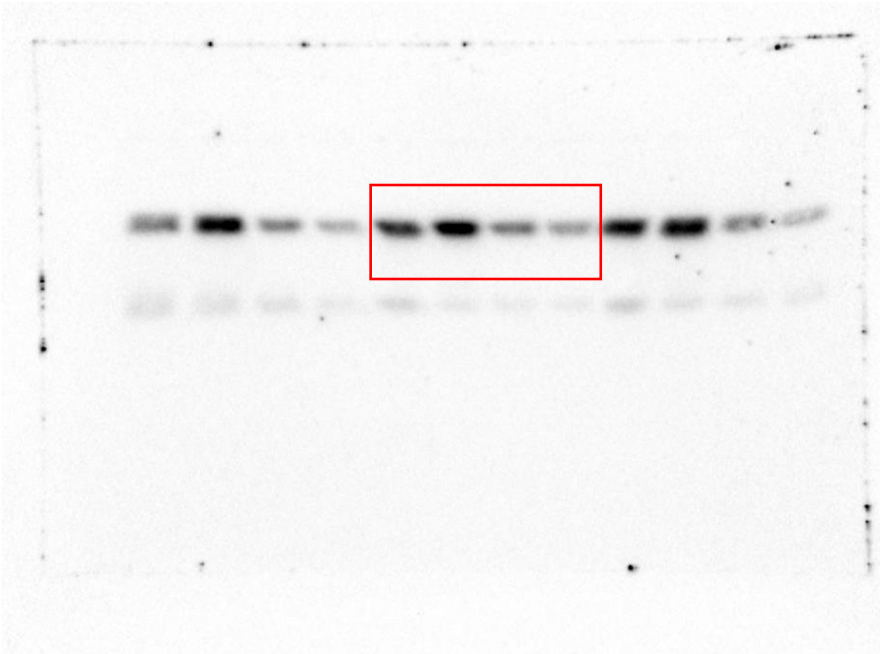

Suppl. Figure 5D TST

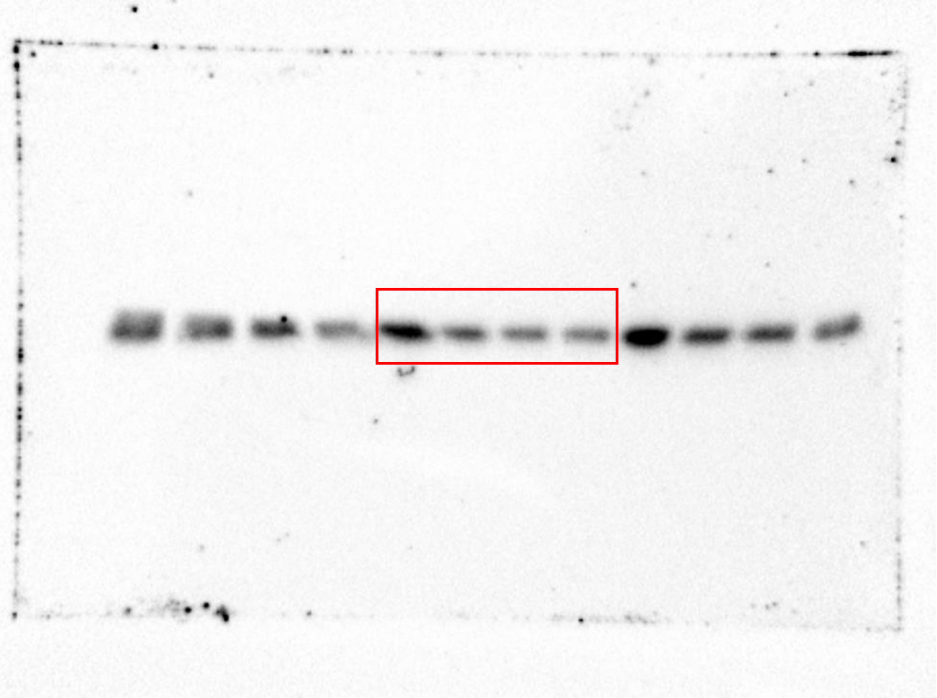

Suppl. Figure 5D SO

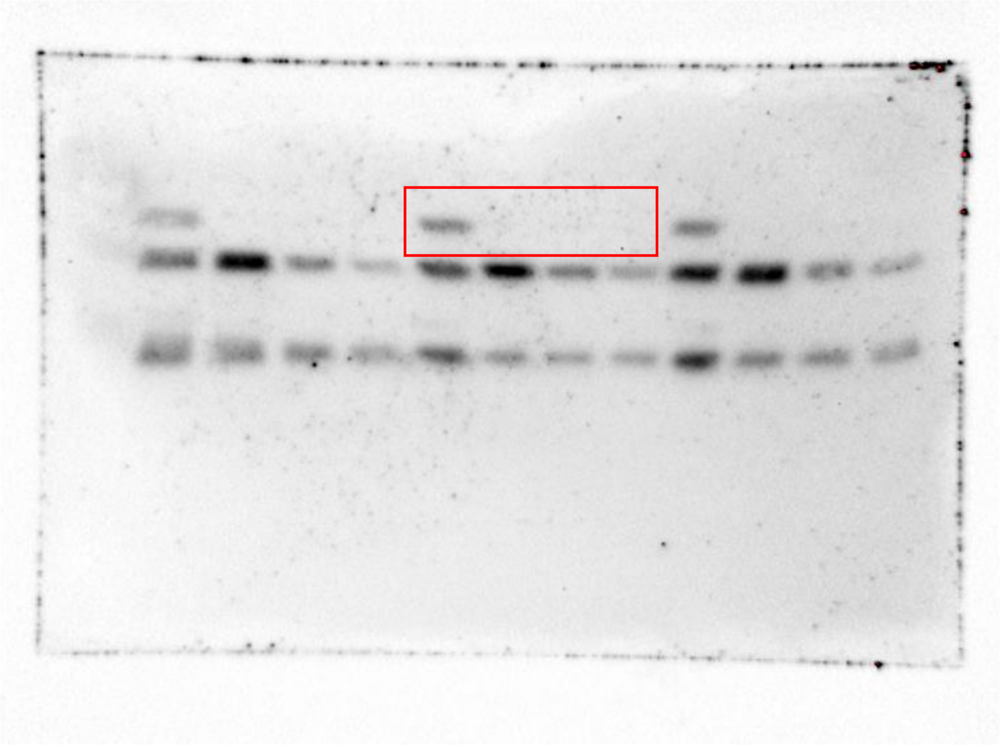

Suppl. Figure 5D VCL

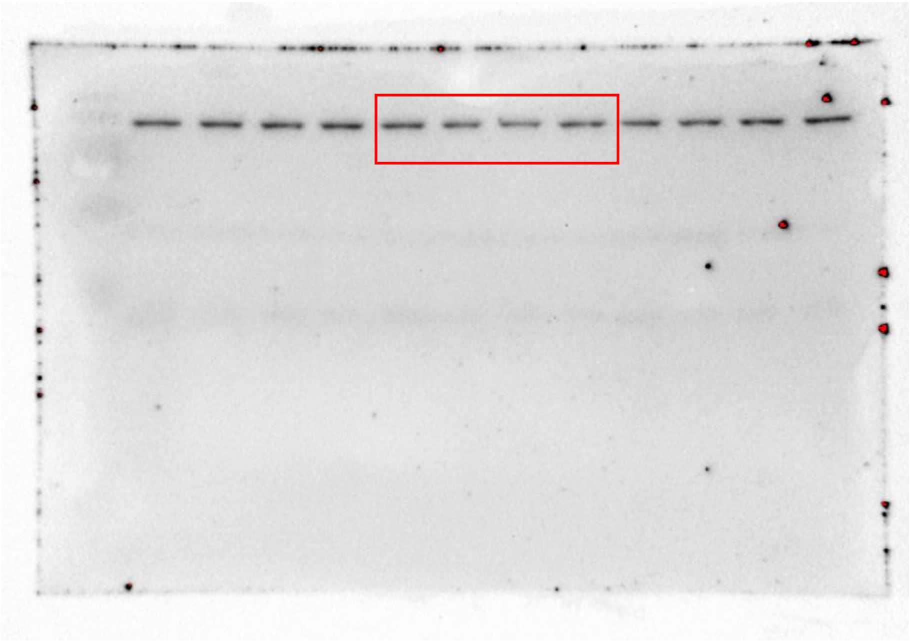

Supplement: Unedited blot and gel images [file jci-135-181299-s328.pdf]
